# SMart Nanoparticle–Hydrogel Hybrid System for Synergistic Eradication of Infection and Promotion of Wound Healing

**DOI:** 10.1002/advs.202517320

**Published:** 2025-11-30

**Authors:** Hongping Wan, Huirong Tan, Xinghong Zhao

**Affiliations:** ^1^ Center for Infectious Diseases Control (CIDC) Sichuan Agricultural University Chengdu 611130 China; ^2^ State Key Laboratory of Veterinary Public Health and Safety College of Veterinary Medicine China Agricultural University Beijing 100193 China

**Keywords:** Bacterial infected wound healing, Bacterial‐targeted photothermal nanoparticles, Fe_2_O_3_‐CS@RBP, multifunctional PAOC‐3@BA hydrogel, Thermostatic photothermal therapy

## Abstract

This study develops an efficient and intelligent photothermal therapy (PTT) system for treating *Staphylococcus aureus*‐infected wounds by integrating a multifunctional hydrogel (PAOC‐3@BA) with *S. aureus*‐targeted photothermal nanoparticles (Fe_2_O_3_‐CS@RBP). The Fe_2_O_3_‐CS@RBP nanoparticles, functionalized with receptor‐binding proteins (RBP) from bacteriophages, selectively recognize *S. aureus* and generate localized heat under near‐infrared (NIR) irradiation for precise bacterial eradication. PAOC‐3 hydrogel, loaded with the anti‐inflammatory agent baicalin (BA), has a cloud point temperature (T_cp_) of ≈47 °C and exhibits strong tissue adhesion and heat‐induced contraction. Upon NIR‐triggered heating, the hydrogel undergoes a turbidity transition, forming a light‐scattering barrier that limits further NIR penetration and maintains a stable therapeutic temperature. This feedback mechanism enhances antibacterial efficacy while preventing tissue overheating. Simultaneously, the hydrogel's high tissue adhesion and contractile behavior promote wound closure and stimulate BA release to suppress inflammation. The system achieves rapid wound healing within one week, offering effective infection control and tissue regeneration. Overall, this work introduces a smart therapeutic platform that integrates bacterial targeting, self‐regulating photothermal therapy, tissue adhesion, and stimulus‐responsive drug release, providing a safe and effective strategy for treating bacterial skin infections.

## Graphical abstract

The integrated system, comprising bacterial‐targeted photothermal nanoparticles (Fe_2_O_3_‐CS@RBP) and multifunctional hydrogels (PAOC‐3@BA) modules, achieves rapid wound healing within one week. This work introduces a smart therapeutic platform that integrates bacterial targeting, self‐regulating photothermal therapy, tissue adhesion, and stimulus‐responsive drug release, providing a safe and effective strategy for treating bacterial skin infections.

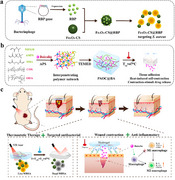

## 1 Introduction

As the body's largest protective organ, the skin serves a vital role in defending against external insults but remains highly susceptible to injury.^[^ ^1^, ^2^, ^3^ ^]^ Infected skin wounds, such as those caused by methicillin‐resistant *S. aureus*, impose a substantial burden on individuals, healthcare systems, and the global economy.^[^ ^4^ ^]^ While antibiotics have traditionally been effective in treating bacterial infections, their widespread misuse and overuse have contributed to the rapid emergence of antibiotic‐resistant strains, posing a major threat to public health.^[^ ^5^, ^6^ ^]^ Consequently, there is an urgent need to develop alternative, non‐antibiotic strategies for the management of *S. aureus* wound infections.^[^ ^7^ ^]^ Moreover, facilitating rapid wound closure is critical, not only to prevent secondary infections, but also to mitigate inflammation and promote tissue regeneration, thereby ensuring timely and effective healing.^[^ ^8^, ^9^ ^]^

Photothermal therapy (PTT) utilizes heat generated by photothermal materials under near‐infrared (NIR) irradiation to inactivate bacteria by disrupting cell membranes and/or denaturing essential proteins.^[^ ^10^, ^11^ ^]^ Owing to its advantages of non‐invasiveness, high selectivity, broad‐spectrum antibacterial activity, and the potential to eliminate drug‐resistant bacteria, PTT has emerged as a promising approach for combating bacterial infections.^[^ ^10^ ^]^ However, conventional PTT systems often lack bacterial specificity and internal temperature‐regulating mechanisms, resulting in reduced antibacterial efficacy and unintended thermal damage to surrounding healthy tissues caused by excessive heat generation.^[^ ^12^ ^]^ Temperatures below 50 °C are generally considered mild for PTT, helping to minimize heat‐induced side effects.^[^ ^13^, ^14^ ^]^ Therefore, there is an urgent need to develop bacteria‐targeted photothermal materials capable of self‐regulating heat output by automatically terminating photothermal conversion upon reaching the optimal therapeutic temperature.

With advances in nanotechnology, numerous nanoparticles with photothermal properties have been developed.^[^ ^15^, ^16^, ^17^, ^18^, ^19^, ^20^ ^]^ However, their clinical translation remains limited, primarily due to the lack of bacterial targeting capability. Bacteriophages,^[^ ^21^, ^22^ ^]^ viruses that specifically infect bacteria, produce receptor‐binding proteins (RBPs) that mediate host recognition and attachment. These RBPs exhibit specificity and affinity that are comparable to those of antibodies. In our previous work,^[^ ^5^, ^6^ ^]^ we successfully synthesized RBPs in vitro using a prokaryotic expression system and demonstrated that RBP‐functionalized nanoparticles, when loaded with antibacterial agents, enable precise and efficient targeted drug delivery. Building on this strategy, we hypothesize that RBP‐modified photothermal nanoparticles could similarly enable targeted bacterial eradication through photothermal mechanisms.

Thermosensitive polymers^[^ ^23^, ^24^ ^]^ with a low critical solution temperature (LCST) are soluble at lower temperatures but undergo a phase transition, typically from linear to spherical structures, when heated above their cloud point temperature (T_cp_), leading to phase separation. Among these, poly(N‐isopropylacrylamide) (PNIPAM, LCST ≈ 33 °C) is widely regarded as a smart material, extensively applied in drug delivery, tissue engineering, and biosensing.^[^ ^25^, ^26^ ^]^ When the temperature exceeds T_cp_, the isopropyl groups in PNIPAM become hydrophobic, forming intramolecular hydrogen bonds that induce gelation, dehydration, and self‐contraction, resulting in an opaque, white structure.^[^ ^12^ ^]^ This transformation generates light‐scattering microdomains, which effectively impede NIR penetration, making PNIPAM a promising material for isothermal photothermal therapy. Nevertheless, PNIPAM hydrogels exhibit poor tissue adhesion and may induce inflammatory responses in infected wounds due to the presence of chemical cross‐linkers, raising concerns about their biosafety

**Scheme 1:**
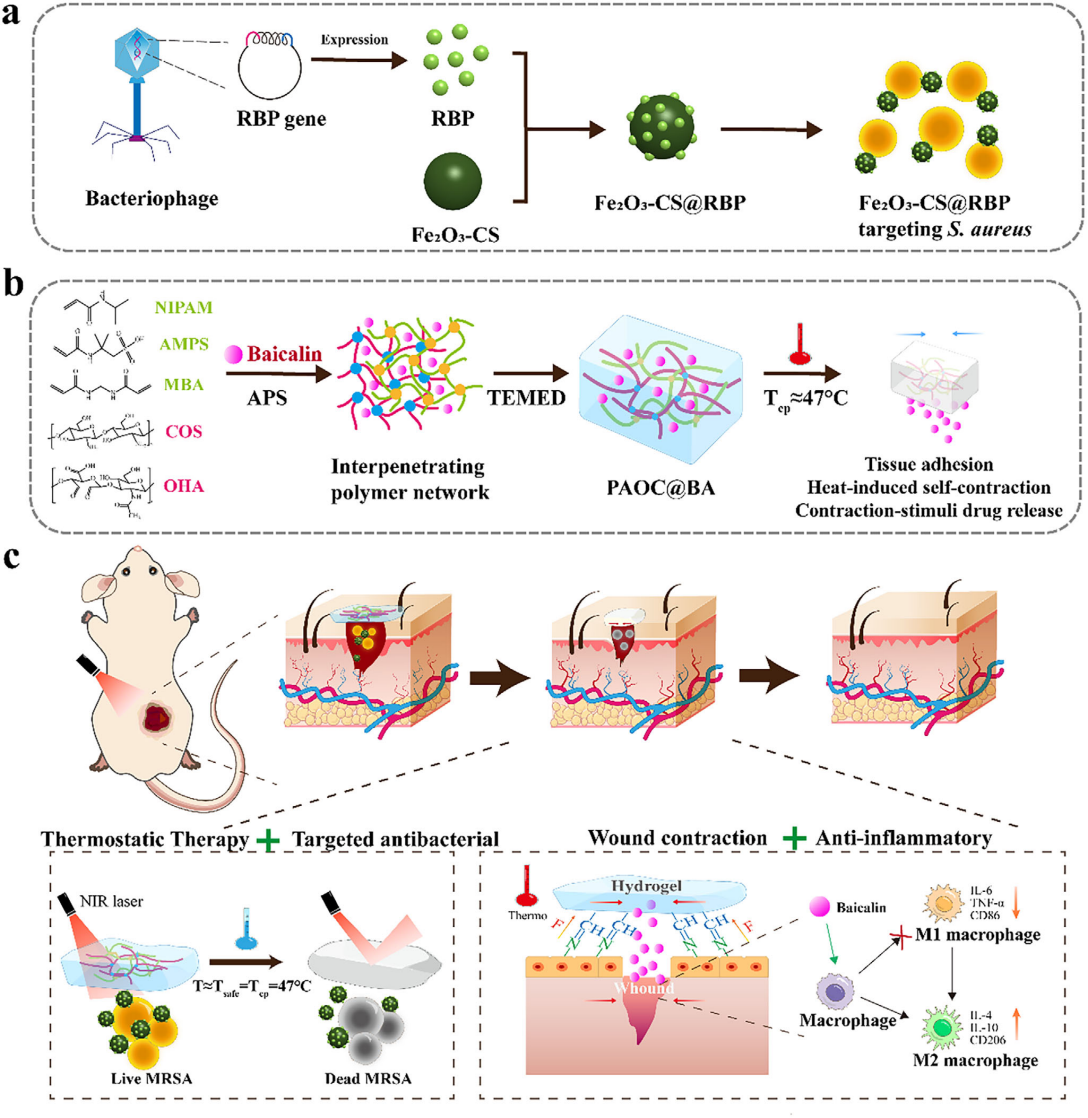
Schematic illustrations of preparation of bacterial‐targeted photothermal nanoparticles (Fe_2_O_3_‐CS@RBP) and multifunctional hydrogels (PAOC‐3@BA), along with their application in promoting the healing of infected wounds. a), Synthesis of bacterial‐ targeted Fe_2_O_3_‐CS@RBP nanoparticles. Upon NIR irradiation, Fe_2_O_3_‐CS@RBP generate localized heat for precise photothermal eradication of the bacteria. b), Fabrication of PAOC‐3@BA hydrogel. PAOC‐3@BA hydrogel exhibiting T_cp_∼47 °C, strong tissue adhesiveness, heat‐induced self‐contraction, and stimuli‐responsive BA release properties. c), The composite Fe_2_O_3_‐CS@RBP and PAOC‐3@BA hydrogel system promotes the *S.aureus*‐infected wound healing. This system provides precise antibacterial activity with minimal thermal damage to healthy tissues. Furthermore, the hydrogel exhibits accelerate wound closure, inflammation suppression, and facilitates tissue regeneration through its multifunctional properties.

Based on the above considerations, we developed a thermosensitive hydrogel‐based PTT platform that combines bacterial‐targeted photothermal nanoparticles with a functional hydrogel matrix. This system provides precise antibacterial activity with minimal thermal damage to healthy tissues. Furthermore, the hydrogel exhibits strong tissue adhesion, heat‐induced contraction, and stimuli‐responsive drug release, all of which contribute to accelerated wound closure, inflammation suppression, and tissue regeneration (**Scheme** **1**). Specifically, RBPs targeting *S. aureus* were expressed using an *Escherichia coli* system and conjugated to chitosan‐modified ferric oxide (Fe_2_O_3_‐CS) photothermal nanoparticles, forming Fe_2_O_3_‐CS@RBP nanoparticles to enhance bacterial specificity. To adjust the T_cp_ of the NIPAM hydrogel to the therapeutic window of 45–50 °C and improve tissue adhesion, NIPAM was copolymerized with 2‐acrylamido‐2‐methyl‐1‐propanesulfonic acid (AMPS). In addition, chitosan oligosaccharide (COS) and oxidized hyaluronic acid (OHA), both known for their biocompatibility and adhesive properties,^[^ ^27^, ^28^, ^29^, ^30^ ^]^ were incorporated to form an interpenetrating polymer network hydrogel (PAOC). Baicalin (BA), a natural compound with anti‐inflammatory properties, was incorporated into the precursor solution of the PAOC to form PAOC@BA. Upon NIR irradiation, Fe_2_O_3_‐CS @RBP nanoparticles selectively target bacteria at the infection site and convert light into thermal energy to eradicate the bacteria. The excessive heat is absorbed by the surrounding PAOC hydrogel, which undergoes a turbidity transition, forming light‐scattering centers that limit further NIR transmission and maintain a stable therapeutic temperature around T_cp_ (47 °C). Concurrently, the hydrogel's tissue‐adhesive and thermos‐responsive contractile properties facilitate wound closure and BA release, further reducing inflammation and enhancing tissue regeneration. To the best of our knowledge, this work presents the first integrated strategy for the safe and effective treatment of bacterial skin infections by combining self‐regulating photothermal therapy, targeted bacterial recognition, tissue adhesion, heat‐induced self‐contraction, and stimuli‐responsive anti‐inflammatory drug release. This work establishes a novel, intelligent, and efficient PTT platform with significant potential for the safe and effective treatment of *S. aureus*‐induced skin infections.

## 2 Results and Discussion

### 2.1 Synthesis and Characterizations of Bacterial‐Targeted Photothermal Module‐ Fe_2_O_3_‐CS@RBP Nanoparticles

To prepare the photothermal agents that can specifically target *S. aureus* to enhance the antibacterial efficiency. We biosynthesized the RBP derived from the staphylococcal bacteriophage, following procedures from our previous work.^[^ ^5^, ^6^ ^]^ Briefly, the gene encoding RBP (Table S1, Supporting Information) was cloned into a plasmid encoding Cys‐6xHis‐GFP tag and expressed in *E. coli* BL21 (DE3) to obtain the thiol‐modified, green fluorescent RBP. After purification by nickel‐affinity chromatography, the RBP was analyzed by sodium dodecyl sulfate‐polyacrylamide gel electrophoresis (SDS‐PAGE) and Western blotting (Wb), confirming the expected size of 79 kDa and high purity (Figure S1, Supporting Information). Next, the *S. aureus*‐specific targeting capability of RBP was assessed by using confocal laser scanning microscopy (CLSM). As shown in Figure S2 (Supporting Information), a strong green fluorescence signal was observed in *S. aureus*, while no fluorescence was detected in *E.coli*, demonstrating the high specificity of RBP for *S. aureus*, consistent with previous findings.^[^ ^5^ ^]^

Iron oxide nanoparticles^[^ ^31^, ^32^, ^33^, ^34^ ^]^ were chosen as the photothermal agents due to their high photothermal conversion efficiency and ease of synthesis route. Therefore, Fe_2_O_3_‐CS was synthesized by one‐step hydrothermal method according to previous work.^[^ ^35^, ^36^ ^]^ The results in Figure S3 (Supporting Information) showed that the obtained Fe_2_O_3_‐CS nanoparticles were sphere with an average size of ≈126.4 ± 0.8 nm and a zeta potential of 17.9 ± 0.9 mV. The Fe_2_O_3_‐CS nanoparticles exhibited excellent photothermal performance. Upon NIR irradiation (808 nm, 1 W cm^−2^), temperature rapidly increased to 80.4 °C at 0.5 mg mL^−1^ within 4 min and stable at 85.2 °C after 8 min irradiation (Figure S4, Supporting Information), aligned with the iron‐based nanomaterials reported by other study.^[^ ^37^, ^38^, ^39^, ^40^, ^41^ ^]^

**Figure 1:**
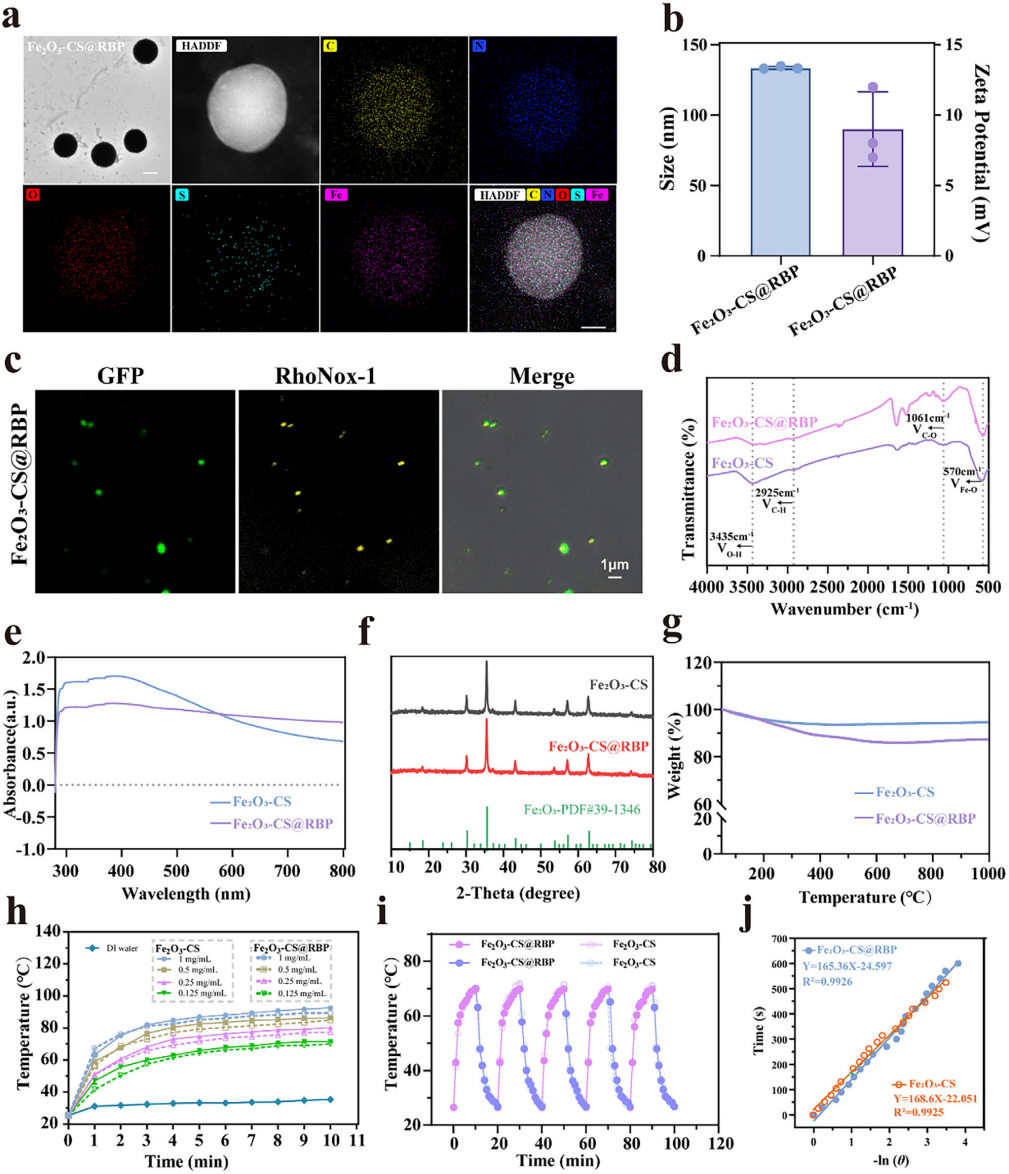
Characterization of bacterial‐targeted photothermal nanoparticles Fe_2_O_3_‐CS@RBP. a), TEM images (Scale bar: 100 nm) and EDS elemental distribution map of Fe_2_O_3_ ‐CS@RBP (Scale bar: 50 nm). b), Average hydrodynamic size and surface zeta‐potential of Fe_2_O_3_‐CS and Fe_2_O_3_‐CS@RBP in ultrapure water, the test results were obtained by dynamic light scattering method. Data are presented as mean ± standard deviation (n = 3 independent experiments). c), CLSM images of Fe_2_O_3_ ‐CS@RBP in which Fe was labeled with RhoNoX‐1 (yellow) and the targeting devices RBP were fused with GFP (green). d), FTIR spectra of Fe_2_O_3_ ‐CS and Fe_2_O_3_‐CS@RBP. e), UV‐vis spectra of Fe_2_O_3_‐CS and Fe2O3‐CS@RBP. f), XRD of Fe_2_O_3_‐CS and Fe_2_O_3_‐CS@RBP. g), Thermogravimetric curves of Fe_2_O_3_‐CS and Fe_2_O_3_‐CS@RBP. h), Photothermal curves of Fe_2_O_3_‐CS and Fe_2_O_3_‐CS@RBP nano‐material irradiated with 808 nm laser at a power density of 1 W cm^−2^ for 10 min. i), Photothermal stability of Fe_2_O_3_ and Fe_2_O_3_‐CS@RBP (0.25 mg mL^−1^) after repeated irradiation at a power density of 1 W cm^−2^ for five times. j), Linear fitting time line and the negative natural logarithm function of the driving force temperature (‐ln θ) obtained from the cooling curves of Fe_2_O_3_‐CS and Fe_2_O_3_‐CS@RBP.

Following confirming of RBP's specificity and binding efficiency, RBP was conjugated to Fe_2_O_3_‐CS nanoparticles by using a heterobifunctional linker, maleimide‐polyethylene glycol‐N‐hydroxysuccinimide (MAL‐PEG_5000_‐NHS), yielding Fe_2_O_3_‐CS@RBP. The morphology of generated Fe_2_O_3_‐CS@RBP were characterized by transmission electron microscopy (TEM). As shown in **Figure** **1a**, Fe_2_O_3_‐CS@RBP retained a spherical morphology. Elemental mapping further confirmed the uniform distribution of carbon, nitrogen, oxygen, iron, and sulfur, across the nanoparticles. Dynamic light scattering (DLS) indicated that zeta potential and the size of Fe_2_O_3_‐CS@RBP was 9 ± 3.9 mV and 133.6 ± 0.3 nm (Figure 1b), consistent with the TEM results. The successful conjugation of RBP to Fe_2_O_3_‐CS was further verified by CLSM. Fe_2_O_3_‐CS was labeled with rhonox‐1 (yellow) and RBP fused with GFP (green). The strong colocalization of both fluorescent signals confirmed effective surface conjugation of RBP. Fourier transform infrared (FTIR) spectra of Fe_2_O_3_‐CS and Fe_2_O_3_‐CS@RBP were shown in Figure 1c. The FTIR spectrum exhibited a distinct Fe─O stretching vibration at 570 cm^−1^. Additionally, bands near 3435 and 1061 cm^−1^ were assigned to O─H and C─O stretching, respectively (Figure 1d). UV–vis absorption spectra (Figure 1e) revealed a broad Fe_2_O_3_ absorption peak^[^ ^42^ ^]^ in both Fe_2_O_3_‐CS and Fe_2_O_3_‐CS@RBP, indicating that RBP conjugation did not alter the core structure of the nanoparticles. X‐ray diffraction (XRD) analysis (Figure 1f) showed diffraction patterns consistent with the standard γ‐ Fe_2_O_3_ phase (PDF No. 39–1346), confirming the crystalline structure of the nanoparticles. Thermogravimetric analysis (TGA) (Figure 1g) demonstrated a higher weight loss in Fe_2_O_3_‐CS@RBP compared to Fe_2_O_3_‐CS with increasing temperature, indicating successful RBP functionalization. The degree of RBP conjugation on the Fe_2_O_3_‐CS surface was estimated to be ≈8.17%.

Next, the photothermal properties of Fe_2_O_3_‐CS@RBP and Fe_2_O_3_‐CS were evaluated at various concentrations and time points under NIR irradiation. As shown in Figure 1h, both materials exhibited comparable photothermal performance at both high (1 mg mL^−1^) and low (0.125 mg mL^−1^) concentrations. The temperature increase was found to be dose‐dependent. At a low concentration (0.125 mg mL^−1^), the temperature rose to ≈70 °C after 10 min of NIR irradiation, whereas at a high concentration (1 mg mL^−1^), it reached up to 92.2 °C under the same conditions. Photothermal stability was evaluated over five heating and cooling cycles. Both Fe_2_O_3_‐CS@RBP and Fe_2_O_3_‐CS maintained consistent temperature profiles, indicating excellent photothermal stability (Figure 1i). The calculated photothermal conversion efficiencies of Fe_2_O_3_‐CS@RBP and Fe_2_O_3_‐CS were approximately 50.7% and 52.9%, respectively (Figure 1j; Figure S4, Supporting Information). These results confirm that both Fe_2_O_3_‐CS@RBP and Fe_2_O_3_‐CS nanoparticles exhibit excellent photothermal activity, and that conjugation with receptor‐binding protein (RBP) does not compromise the photothermal performance of the Fe_2_O_3_‐CS nanoparticles.

### 2.2 In Vitro Bacterial Targeting Ability and Photothermal Antibacterial Activity of Fe_2_O_3_‐CS@RBP

**Figure 2:**
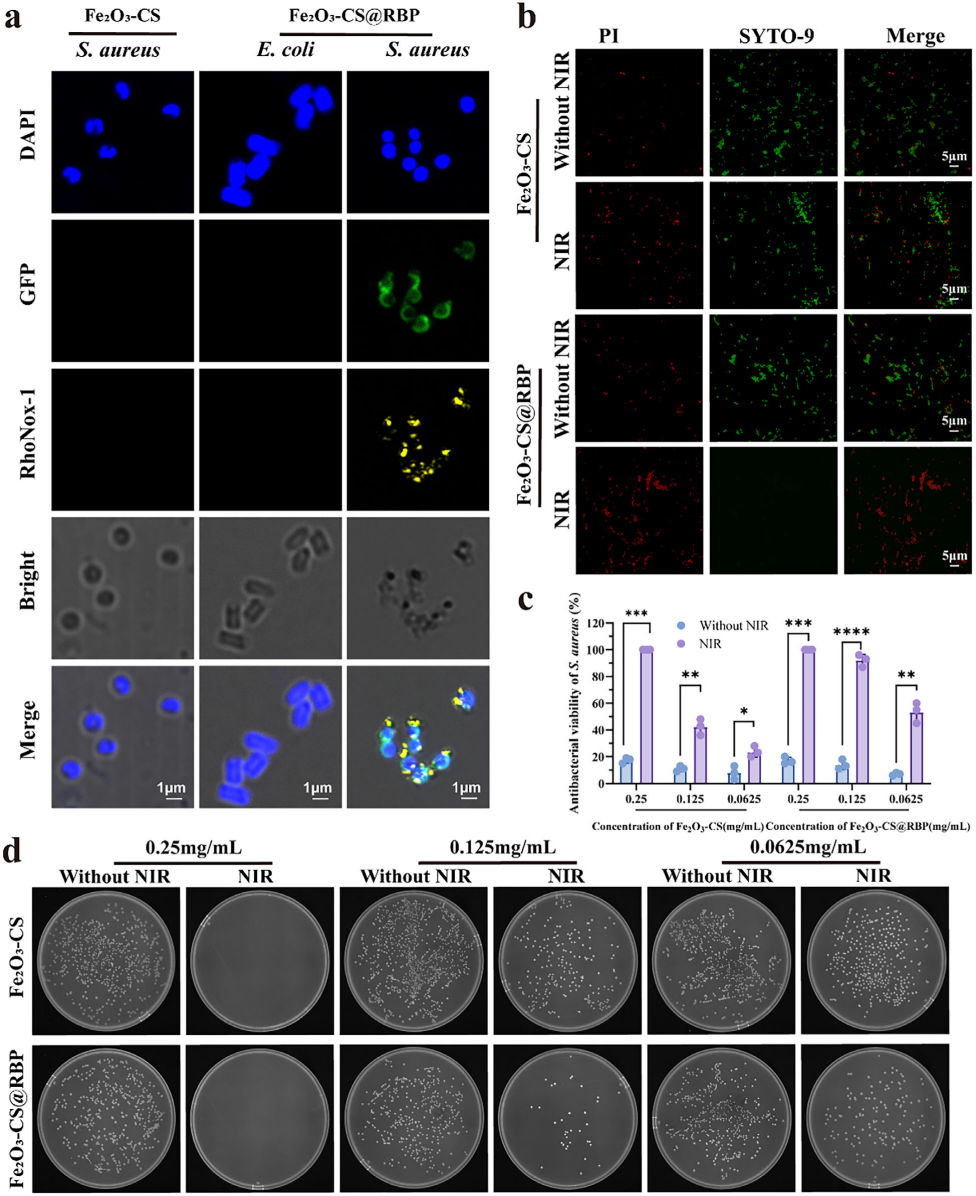
Bacterial targeting ability and photothermal antibacterial activity of Fe_2_O_3_‐CS@RBP. a), CLSM images of *MRSA* and *E. coli* after incubation with Fe_2_O_3_‐CS or Fe_2_O_3_‐CS@RBP, in which the Fe was labeled with RhoNoX‐1 (yellow) and the targeting RBP were fused with GFP (green). Bacteria nucleoid was stained with DAPI (blue). Experiment was repeated three times with similar results. b), Fluorescence micrographs of live (green) and dead (red) bacteria in the Fe2O3‐CS and Fe2O3‐CS@RBP (0.25 mg mL^−1^) groups without and with NIR irradiation. c‐b), Photographs and statistical analysis of bacterial colonies formed on agar plates for different concentrations of Fe_2_O_3_‐CS and Fe_2_O_3_‐CS@RBP with or without NIR irradiation (808 nm, 1 W cm^−2^, 10 min). Data presented as mean ±standard deviation (n = 3 independent experiments). The statistical significance of the data was assessed using one‐way ANOVA followed by Tukey's multiple comparisons test. *p < 0.05 **p < 0.01, ***p < 0.001 compared with the without NIR group.

To evaluate the in vitro efficacy of the Fe_2_O_3_‐CS@RBP nanoparticles, their specific targeting capability toward *S‐aureus* and their photothermal antibacterial activity NIR irradiation were systematically investigated. First, the *S. aureus* targeting capability of Fe_2_O_3_‐CS@RBP was performed by CLSM assay. Rhonox‐1‐labeled (yellow) Fe of Fe_2_O_3_‐CS@RBP was incubated with DAPI‐stained *S. aureus*. The microscopy images (**Figure** **2a**) revealed strong colocalization between Fe_2_O_3_‐CS@RBP and *S. aureus*, indicating efficient and specific binding. In contrast, no binding was observed with *E. coli* or with Fe_2_O_3_‐CS alone, confirming that the targeting ability is mediated by the RBP component. These findings demonstrate that Fe_2_O_3_‐CS@RBP possesses strong specificity for *S. aureus*, supporting its potential for targeted antibacterial therapy at infection sites. The antibacterial effect of Fe_2_O_3_‐CS@RBP and Fe_2_O_3_‐CS under NIR irradiation was first investigated by CLSM assay on live and dead staining of bacteria (Figure 2b).

Following confirmation of the bacterial targeting ability of Fe_2_O_3_‐CS@RBP, we next evaluated its antibacterial activity at various concentrations under NIR irradiation. As shown in Figure 2b, compared to Fe‐CS, lots of *S. aureus* death (red) was observed under CLSM assay after Fe_2_O_3_‐CS@RBP with NIR treatment. In addition, Fe_2_O_3_‐CS@RBP exhibited excellent antibacterial activity, which was found to be dose‐dependent (Figures 2c,d; Figure S5, Supporting Information). At a concentration of 0.25 mg mL^−1^, complete bacterial eradication (100% killing efficiency) was achieved under NIR irradiation. Even at a lower concentration of 0.125 mg mL^−1^, the antibacterial rate remained above 90%. In contrast, Fe_2_O_3_‐CS alone demonstrated significantly lower efficacy at the same concentration, with an antibacterial efficiency of less than 45%. These results indicate that the RBP‐mediated targeting enhances the antibacterial performance of the photothermal nanomaterial. This enhancement is likely due to the selective accumulation of nanoparticles at the bacterial site, which leads to increased local temperature under NIR irradiation and thereby more efficient bacterial eradication.

### 2.3 Synthesis and Characterizations of Multifunctional Intelligent Hydrogel Module:PAOC‐3@BA Hydrogel

To synthesize a multifunctional hydrogel with self‐regulating photothermal activity, tissue adhesion, heat‐induced self‐contraction, and stimuli‐responsive anti‐inflammatory drug release capabilities, N‐isopropylacrylamide (NIPAM) was copolymerized with 2‐acrylamido‐2‐methyl‐1‐propanesulfonic acid (AMPS) to adjust the hydrogel's T_cp_ to the therapeutic window of 45–50 °C. To enhance adhesive properties, COS and OHA (oxidation degree of 38.19%, Figure S6, Supporting Information) were incorporated to construct an interpenetrating polymer network, forming the PAOC hydrogel. Hydrogels with varying COS concentrations were prepared and designated as PAOC (0 mg mL^−1^), PAOC‐1 (0.1 mg mL^−1^), PAOC‐2 (0.5 mg mL^−1^), and PAOC‐3 (1 mg mL^−1^), respectively. Furthermore, baicalin (BA), a natural anti‐inflammatory compound, was incorporated into the hydrogel precursor solution to obtain the drug‐loaded hydrogel, named PAOC@BA, PAOC‐1@BA, PAOC‐2@BA, and PAOC‐3@BA.

**Figure 3:**
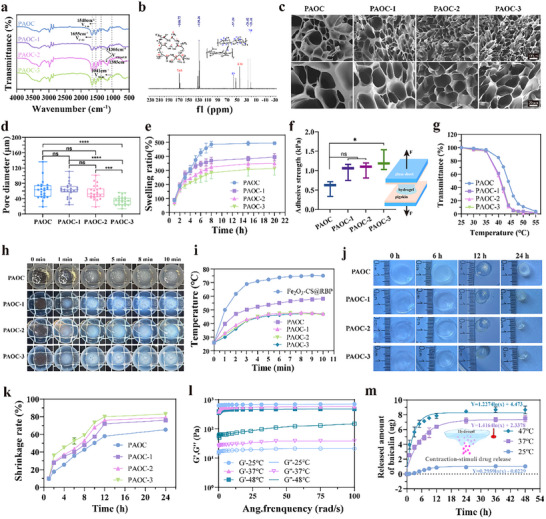
Characterization of PAOC‐3 hydrogel. a), FTIR spectrum of PAOC hydrogel, where PAOC, PAOC‐1, PAOC‐2, and PAOC‐3 were synthesized using the same method, with COS contents of 0, 0.1, 0.5, and 1 mg mL^−1^, respectively. b), Solid‐state ^13^C NMR spectrum of PAOC‐3 hydrogel. c‐d), Scanning electron microscope images (c) of the solid‐state of different hydrogels and the measured pore size (d) of the hydrogel. Data presented as mean ± standard deviation (n = 16). e), Real‐time swelling rate of PAOC hydrogel within 20 h at 25 °C. Data presented as mean ± standard deviation (n = 3) f), Adhesive strength of different groups of hydrogels with consistent initial size at 25 °C. Data presented as mean ± standard deviation (n = 3 independent experiments). g), Transmittance of the PAOC, PAOC‐1, POC‐2 and PAOC‐3 hydrogels at 808 nm as a function of temperature. h), Photographs showing the change in the appearance of different groups under the presence of Fe_2_O_3_‐CS@RBP (0.5 mg mL^−1^) and after being irradiated by 808 nm laser with a power density of 1 W cm^−2^ to cause phase‐transition in the hydrogels. i), Photothermal curves of different groups of hydrogels after being irradiated by 808 nm near‐infrared light (1 W cm^−2^) with Fe_2_O_3_‐CS@RBP (0.5 mg mL^−1^) incorporated. j), Photographs of the self‐contraction of PAOC, PAOC‐1, PAOC‐2, and PAOC‐3 hydrogels (6 mm in diameter,4 mm in thickness) under thermal stimulation at 37 °C for 24 h. k), The shrinkage rate of the PAOC, PAOC‐1, PAOC‐2 and PAOC‐3 hydrogels as a function of time at 37 °C. Data presented as SD ± average value (n = 3 independent experiments). l), Rheological properties of PAOC‐3 hydrogel at different temperatures (25, 37, and 48 °C) as a function of frequency (0.1–100 rad s^−1^). m), The release curve of BA by the PAOC‐3@BA hydrogel at a constant temperature of 37 °C. The data is presented as the mean ± standard deviation (n = 3 independent experiments). The statistical significance of the data was assessed using one‐way ANOVA followed by Tukey's multiple comparisons test. ns, no significance; *p < 0.05, ***p < 0.001, ****p < 0.0001.

FTIR spectroscopy (**Figure** **3a**) demonstrated characteristic peaks corresponding to the isopropyl group (1366 and 1385 cm^−1^) and sulfonic acid group (‐SO_3_H, 1041 cm^−1^),^[^ ^43^, ^44^ ^]^ confirming the successful incorporation of NIPAM and AMPS into the hydrogel network. Additional absorption peaks at 1655 cm^−1^ (C═O stretching, amide I) and 1540 cm^−1^ (N─H bending, amide II) indicated the presence of amide groups.^[^ ^45^, ^46^ ^]^ Solid‐state ^13^C NMR spectra (Figure 3b; Figure S7, Supporting Information) further verified the structural composition of all four hydrogel groups.

Scanning electron microscopy (SEM) was used to observe the microstructure difference in all four hydrogels. As shown in Figure 3c, all hydrogels exhibited homogeneous, interconnected 3D porous structures. Nevertheless, the pore size (Figure 3d) varied significantly in PAOC (63.3 ± 41.9 µm), PAOC‐1 (63.1 ± 30.3 µm), PAOC‐2 (56.7 ± 29.3 µm), and PAOC‐3 (34.1 ± 18.2 µm). It can be inferred that the pore size of the PAOC hydrogels is closely correlated with the COS concentration: as the COS concentration increases, the pore size of the hydrogel decreases. This phenomenon is primarily attributed to the formation of a denser cross‐linked network between COS and OHA, which reinforces the NIPAM‐AMPS polymer matrix and restricts the network expansion, resulting in smaller pores.

The swelling and adhesion behaviors of hydrogels were evaluated (Figure 3e,f). All hydrogels reached the maximum expansion rate within 8 h. Increasing COS concentration led to reduced swelling ratios, consistent with SEM results, confirming enhanced molecular entanglement and cross‐linking density. The tissue adhesive of each hydrogel was quantificationally measured using the modified tensile testing method (Figure 3f). The PAOC‐3 exhibited the highest tissue adhesive strength (1.25 ± 0.28 kPa), compared to PAOC (0.56 ± 0.21 kPa), PAOC‐1 (0.99 ± 0.22 kPa), and PAOC‐2 (1.04 ± 0.20 kPa). This enhanced adhesion can be attributed to the presence of COS, which contributes to the tissue‐adhesive properties of the formulation. The strong adhesion is further enhanced by the formation of hydrogen bond between COS and tissue, as well as the formation of dynamic Schiff‐base linkages between OHA and tissue amine groups. These synergistic interactions significantly enhance tissue adhesion. High‐adhesion bio‐glues or hydrogels that firmly adhere to the wound surface play a critical role in preventing secondary infections and thus provide substantial benefits for the wound healing process.^[^ ^47^, ^48^, ^49^ ^]^ The strong tissue adhesion exhibited by the PAOC‐3 hydrogel may enhance its ability to maintain intimate contact with the wound bed, creating a protective barrier against bacterial invasion and supporting tissue regeneration.

Next, we assessed the capability of theses hydrogels on regulating the transmittance of NIR light during phase transition. Briefly, a hydrogel disc was placed atop a cuvette filled with Fe_2_O_3_‐CS@RBP solution (0.5 mg mL^−1^) to make them physically contact with each other, formed a sealed interface. Then an 808 nm NIR laser (1 W cm^−2^) was irradiated at a direction perpendicular to the hydrogel. The photothermal conversion of Fe_2_O_3_‐CS@RBP elevated the system temperature, triggering a visible phase transition in the hydrogel from transparent to opaque white to reduce NIR light penetration (Figure 3g,h), thereby establishing an automatic photothermal feedback loop. The transmittance of the PAOC‐1, PAOC‐2, and PAOC‐3 hydrogels at 808 nm was measured as a function of temperature to determine the T_cp_, defined at 5% transmittance^[^ ^12^ ^]^ (Figure 3g). Upon reaching T_cp_, the irradiated area of the hydrogel became visibly opaque, effectively blocking further NIR transmission and stabilizing the temperature near T_cp_ and preventing overheating. In the absence of PAOC hydrogel, the photothermal temperatures finally stabilized close to 60 °C after irradiation for 10 min. In contrast, the PAOC‐1, PAOC‐2, and PAOC‐3 hydrogels can maintain a stable photothermal temperature ≈ 47 °C (Figure 3i), suggesting the feasibility of using PAOC‐1, PAOC‐2 and PAOC‐3 for safe and precise temperature control. Mild PTT, typically conducted at temperatures below 50 °C, has attracted increasing clinical interest due to its ability to effectively eradicate bacteria while minimizing damage to surrounding healthy tissues. In clinical practice, achieving mild PTT often requires careful adjustment of multiple experimental parameters, including the concentration of photothermal agents, laser power density, and irradiation duration.^[^ ^50^, ^51^, ^52^, ^53^ ^]^ However, this process is often cumbersome and lacks universality, limiting its practical application and reproducibility in diverse clinical settings. The PAOC‐1, PAOC‐2, and PAOC‐3 hydrogels exhibit intelligent and precise temperature regulation by modulating the transmittance of near‐infrared (NIR) light. This self‐regulating capacity enables stable maintenance of therapeutic temperatures within the mild PTT range, offering a promising strategy to simplify treatment protocols and enhance the safety and efficacy of clinical photothermal applications.

The thermo‐stimulated auto‐shrinkage behavior of each hydrogel was measured at 37 °C to mimic the in vivo situation. Hydrogels of PAOC‐1, PAOC‐2, PAOC‐3 exhibited significantly higher shrinkage rates compared to PAOC, with substantial shrinkage observed within the first 12 h (Figure 3j,k). These results support the auto‐shrinkage behavior driven by the lower critical solution temperature (LCST) of PNIPAM (≈33 °C), above which the hydrogel network transitions from a hydrophilic to a hydrophobic state, leading to dehydration and volume reduction. Shrinkage behavior of wound dressing important for the wound closure that can accelerate the wound healing process. Hydrogels of PAOC‐1, PAOC‐2, and PAOC‐3 exhibited notable thermo‐induced shrinkage at 37 °C. This contraction behavior mimics the natural wound contraction mechanism, potentially drawing wound edges closer together and facilitating faster tissue regeneration. Therefore, the enhanced shrinkage capacity of PAOC‐1, PAOC‐2, and PAOC‐3 hydrogels may offer substantial benefits for improving wound healing outcomes.

Among the tested formulations, PAOC‐3 exhibited the highest tissue adhesion, the largest thermos‐induced shrinkage rate, and stable self‐regulation of photothermal temperature within the safe therapeutic range (≈47 °C). Therefore, PAOC‐3 was selected for subsequent investigations. Initially, the impact of hydrogel thickness on NIR penetration and system temperature was explored. As shown in Figure S8 (Supporting Information), with PAOC‐3 thickness ranging from 2 to 4 mm, the system temperature remained approximately at 47 °C. However, with an increased thickness of 8 mm, the temperature stabilized below 44 °C. This phenomenon is attributed to the greater thickness of the hydrogel, leading to reduced NIR penetration and consequently lowering the system temperature.

The stiffness and flexibility of hydrogels are able to significantly regulate cell’ behavior. Therefore, the viscoelastic behavior of PAOC‐3 was analyzed using a rheometer. As shown in Figure 3l, at 25, 37, and 48 °C, the storage modulus (G′) consistently exceeded the loss modulus (G″) across a frequency range of 0.1–100 rad s^−1^, indicating stable gel‐like behavior and mechanical integrity under physiological and photothermal conditions. After confirming the self‐regulating photothermal therapy, strong tissue adhesion, heat‐induced self‐contraction capabilities, BA was loaded into PAOC‐3 to create PAOC‐3@BA by incorporating BA into the precursor solution. Drug release studies demonstrated negligible BA release at 25 °C, whereas sustained release profile occurred at 37 and 47 °C (Figure 3m). The release behavior closely follows a first‐order kinetic model, characterized by a rapid initial release followed by a slower, sustained release phase. This release profile is advantageous for maintaining effective drug concentrations over time. The drug release behavior under NIR irradiation was also investigated. As shown in Figure S9 (Supporting Information), NIR irradiation alone did not stimulate the release of BA. However, when photothermal particles (Fe_2_O_3_‐CS@RBP) were introduced, a detectable amount of BA was released. This suggests that the localized heat generated by the photothermal particles induced hydrogel shrinkage, thereby triggering the release of BA (Figure S9, Supporting Information). This could be attribute to the thermo‐stimulated shrinkage behavior leading to dehydration occurred and BA was taken out. The results suggest PAOC‐3@BA hydrogel have stimuli‐responsive BA release capabilities. Importantly, incorporation of BA did not significantly alter the hydrogel's physicochemical properties of all hydrogel formulations, including morphology, swelling ratio, rheological behavior, adhesive strength, shrinkage capacity, and photothermal responsiveness (Figures S10–S16, Supporting Information). This suggests that BA is not chemically bound within the hydrogel network and can be freely released during thermo‐stimulated shrinkage process of hydrogel. These results also indicate the hydrogel's potential as a versatile drug delivery platform for other therapeutic agents.

### 2.4 In Vitro Photothermal‐Antibacterial and Anti‐Inflammatory Activity of the Combined Intelligent Hydrogel (PAOC‐3@BA) and Bacterial‐Targeted Photothermal Nanoparticles (Fe_2_O_3_‐CS@RBP) System

**Figure 4:**
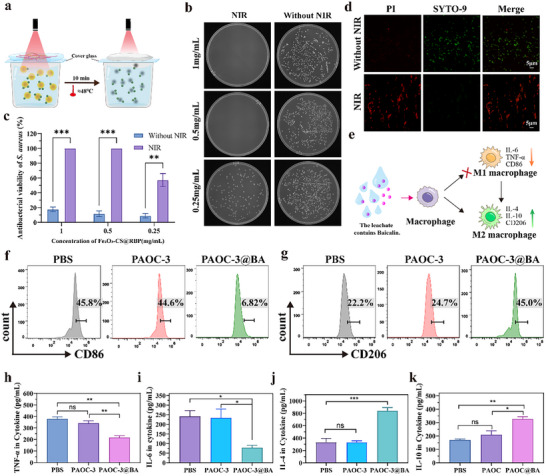
Photothermal‐antibacterial and anti‐inflammatory activity of the combined intelligent hydrogel (PAOC‐3@BA) and bacterial‐targeted photothermal nanoparticles (Fe_2_O_3_‐CS@RBP) in vitro. a), The schematic diagram of composite system by Fe_2_O_3_‐CS@RBP and PAOC‐3@BA hydrogel. b), Photographs of bacterial colonies formed on agar plates from the composite system with different concentration of Fe_2_O_3_‐CS@RBP, with or without 808 nm NIR (1 W cm^−2^) treatment. c), Antibacterial activity of the composite system with different concentration of Fe_2_O_3_‐CS@RBP, with or without 808 nm NIR (1 W cm^−2^) treatment. Data are presented as mean ± standard deviation (n = 3 biological replicates). The statistical significance of the data was assessed using one‐way ANOVA followed by Tukey's multiple comparisons test. **p < 0.01, ***p < 0.001 compared with the without NIR group. d), Fluorescence micrographs of live (green) and dead (red) bacteria in the composite system with Fe_2_O_3_‐CS@RBP (0.5 mg mL^−1^) groups with or without NIR irradiation. e), Anti‐inflammatory schematic diagram of PAOC‐3@BA hydrogel. f,g), The ratio of M1 (f) and M2 (g) macrophages was measured by flow cytometry analysis. CD86 stimulated the surface pro‐inflammatory M1 phenotype of macrophages. CD206 stimulated macrophages, indicating the anti‐inflammatory M2 phenotype. Experiment was repeated three times with similar results. h–k), The expression levels of cytokines after the leaching solution of the PAOC‐3@BA hydrogel acted on RAW264.7 macrophages. TNF‐α (h); IL‐6(i); IL‐4(g); IL10(k). Data are presented as mean ± standard deviation (n = 3 independent experiments). The statistical significance of the data was assessed using one‐way ANOVA followed by Tukey's multiple comparisons test. ns, no significance, *p < 0.05. **p < 0.01, ***p < 0.001.

Next, we investigated the in vitro antibacterial performance of the composite system comprising the intelligent hydrogel (PAOC@BA) and the *S. aureus‐* targeted photothermal antibacterial nanoparticles (Fe_2_O_3_‐CS@RBP). The antibacterial efficacy was evaluated against the clinically relevant methicillin‐resistant *S. aureus* (MRSA) using varying concentrations of Fe_2_O_3_‐CS@RBP (1.0, 0.5, and 0.25 mg mL^−1^), both in the absence and presence of NIR irradiation. As shown in **Figure** **4a–c**, in the absence of NIR irradiation, the composite system exhibited limited antibacterial activity, with bacterial survival rates remaining near 90%, consistent with the activity of Fe_2_O_3_ reported by other researches.^[^ ^54^ ^]^ In contrast, upon NIR irradiation, a clear concentration‐dependent antibacterial effect was observed. At Fe_2_O_3_‐CS@RBP concentrations of 1.0 and 0.5 mg mL^−1^, bactericidal efficiency reached 100%, while at 0.25 mg mL^−1^, the efficiency was 57.3%. These results corresponded to photothermal equilibrium temperatures of ≈47.6, 47.2, and 41.0 °C, respectively, as shown in Figure S17 (Supporting Information). The potent antibacterial performance of the composite system was further validated using fluorescence microscopy at a Fe_2_O_3_‐CS@RBP concentration of 0.5 mg mL^−1^ (Figure 4d). Results revealed extensive bacterial death, as indicated by strong red fluorescence, thereby confirming the effective bactericidal activity of the Fe_2_O_3_‐CS@RBP nanoparticles under NIR irradiation.

Inflammation is a critical component throughout the wound healing process. Macrophages, key players in the innate immune response, are abundant in inflamed tissues and exist primarily in two phenotypes: pro‐inflammatory M1 and anti‐inflammatory M2.^[^ ^55^, ^56^, ^57^ ^]^ M1 macrophages secrete pro‐inflammatory cytokines such as TNF‐α and IL‐6, while M2 macrophages produce anti‐inflammatory cytokines, including IL‐10 and IL‐4.^[^ ^55^ ^]^ Upon NIR irradiation, temperature‐induced hydrogel shrinkage and dehydration, facilitating BA release. BA, a flavonoid compound, has been demonstrated to possess anti‐inflammatory properties (Figure 4e). Therefore, the leaching solution from the hydrogel was incubated with lipopolysaccharides (LPS)‐stimulated macrophage (RAW264.7) for 24 h, followed by flow cytometry and ELISA analysis. As shown in Figure 4f,g, significantly lower percentage of M1 macrophages (labeled by CD86) was observed in PAOC‐3@BA (6.82%) than that of PAOC‐3 (44.6%). Conversely, higher percentage of M2 macrophages (labeled by CD206) was observed in PAOC‐3@BA (45.0%) than that in PAOC‐3 (24.7%). In addition, significantly lower of proinflammatory cytokines TNF‐α (Figure 4h) and IL‐6 (Figure 4i), along with higher levels of anti‐inflammatory cytokines IL‐4 (Figure 4j) and IL‐10 (Figure 4k), were observed in PAOC‐3@BA compared to the PAOC‐3. These results demonstrated the BA released from PAOC‐3@BA effectively promotes macrophage polarization from the M1 to the M2 phenotype, which is advantageous for resolving inflammation and accelerating wound healing.

The biocompatibility of PAOC‐3@BA and Fe_2_O_3_‐CS@RBP was evaluated both in vitro and in vivo. The biosafety of these materials was first assessed via a hemolysis assay. As shown in Figure S18a (Supporting Information), no hemolytic activity was observed either the PAOC‐3@BA hydrogel or the Fe_2_O_3_‐CS@RBP nanoparticles even at concentrations as high as 2 mg mL^−1^, indicating excellent hemocompatibility of both components. In vitro cytocompatibility was further confirmed through co‐culture with Vero cells, HepG2 cells, and HEK‐293T cells. Cell viability in all treatment groups exceeded 90% (Figure S18b–e, Supporting Information) even the concentration of nanoparticles up to 2 mg mL^−1^, demonstrating that both Fe_2_O_3_‐CS@RBP and PAOC‐3@BA possess high biocompatibility and low cytotoxicity. Furthermore, the release of iron content from the nanoparticles after incubation with DMEM, with or without NIR treatment, was less than 1% (Table S2, Supporting Information), highlighting the stability of Fe_2_O_3_‐CS@RBP and Fe_2_O_3_‐CS nanoparticles. In vivo biocompatibility was additionally validated by applying the materials to wound surfaces in a mouse model. Histological examination revealed no pathological changes in the spleen, with tissue architecture remaining intact. Furthermore, no abnormalities were observed in the heart, lungs, liver, or kidneys (Figure S19, Supporting Information), further confirming the systemic biosafety of PAOC‐3@BA and Fe_2_O_3_‐CS@RBP.

### 2.5 The Combined Module of Intelligent Hydrogel (PAOC‐3@BA) and Bacterial‐Targeted Photothermal Nanoparticles (Fe_2_O_3_‐CS@RBP) Effectively Accelerates the Infectious Wounds Healing

**Figure 5:**
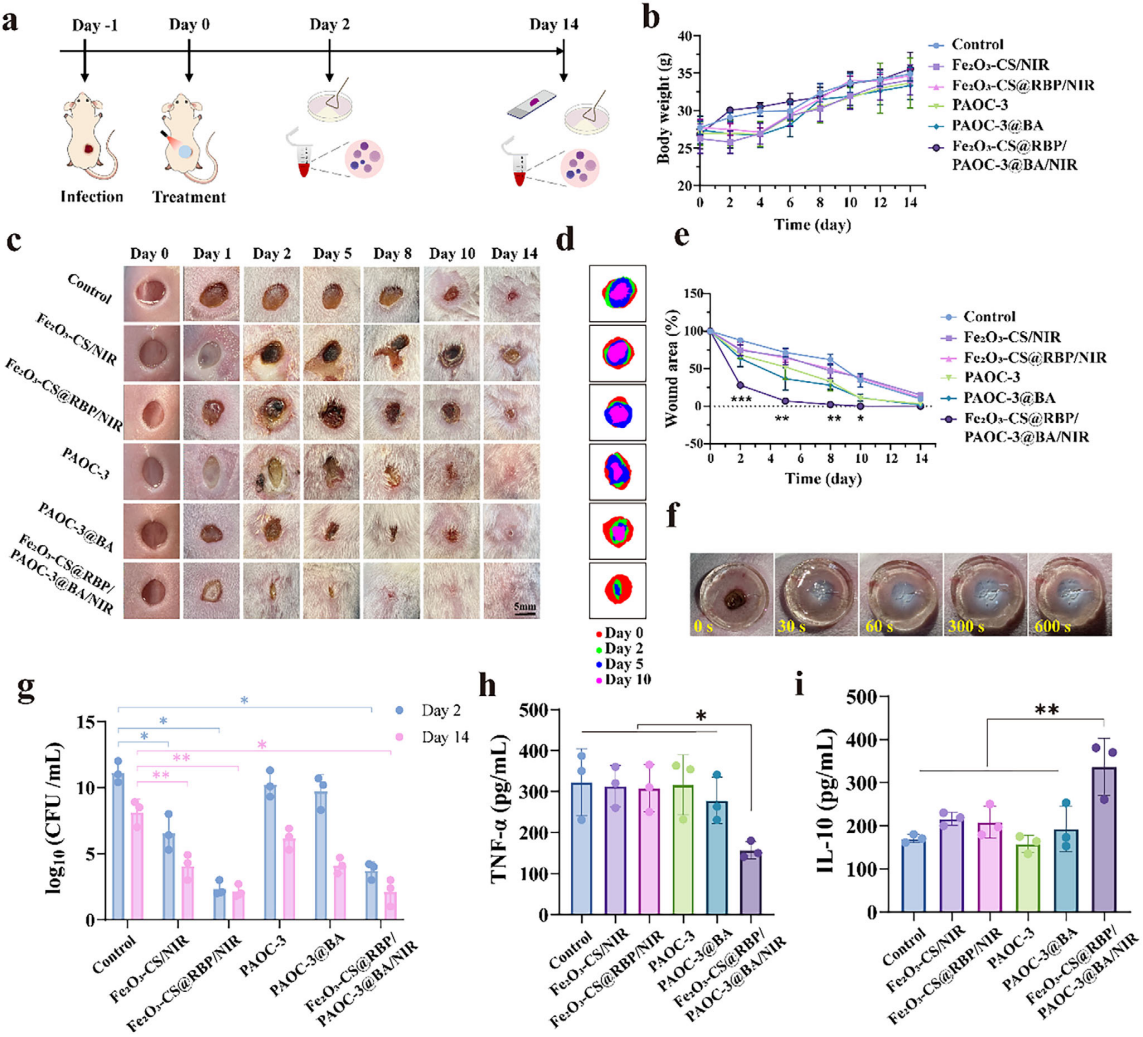
The combined module of intelligent hydrogel (PAOC‐3@BA) and bacterial‐targeted photothermal nanoparticles (Fe_2_O_3_‐CS@RBP) effectively accelerates the infectious wounds healing. a), The treatment experimental plan for the mouse wound infection model. b), The body weights of mice under different treatments. Data are presented as mean ± standard deviation (n = 5 biological replicates). c), Photos of infected wounds treated with different methods. Scale: 5 mm. d), Schematic diagram illustrating the healing process of wounds under different treatments by using image J. e), Quantitative analysis of the wound areas in mice under different treatments. Data are presented as mean ± standard deviation (n = 3 biological replicates). f), Photo of the phase transition process of PAOC@BA hydrogel during the thermostatic photothermal therapy with 808 nm laser (1 W cm^−2^) irradiation of MRSA‐infected wounds in mice. g), The bacterial viability of the skin tissue on the 2nd and 14th post‐treatment days was statistically analyzed using log10 (CFU mL^−1^). Data are presented as mean ± standard deviation (n = 3 biological replicates). h‐I), The expression levels of TNF‐α (h) and IL‐10 (i) in the mouse skin tissues on the 14th day were quantitatively analyzed by ELISA. Data are presented as mean ± standard deviation (n = 3 biological replicates). The statistical significance of the data was assessed using one‐way ANOVA followed by Tukey's multiple comparisons test, *p < 0.05. **p<0.01.

Motivated by the promising in vitro antibacterial results and excellent biocompatibility, an animal model of *S. aureus*‐infected wounds was established to evaluate the in vivo therapeutic efficacy of the combined system. In this system, Fe_2_O_3_‐CS@RBP (0.5 mg mL^−1^) generate localized heat upon 10 min of NIR irradiation, enabling precise photothermal eradication of *S. Aureus*. Simultaneously, PAOC‐3@BA keep constant and safety wound temperature, while offering high tissue adhesiveness, heat‐induced self‐contraction, and stimuli‐responsive release of anti‐inflammatory drug, accelerating the wound healing process. To verify this hypothesis, *S. aureus*‐infected wound‐bearing mice were randomly divided into six groups: Control, Fe_2_O_3_‐CS/NIR, Fe_2_O_3_‐CS@RBP/NIR, PAOC, PAOC@BA, Fe_2_O_3_‐CS@RBP/PAOC‐3@BA/NIR. Each group received the corresponding treatment as illustrated in **Figure** **5a**. During the treatment, the changes in body weight and wound size were recorded at predetermined time points. All groups exhibited a gradual increase in body weight with no significant differences, indicating the biosafety of all treatment materials (Figure 5b).

Representative photographs of wounds at various treatments and time points (Figure 5c,d) revealed that all treatment groups showed progressive wound contraction. Notably, the wound treated with Fe_2_O_3_‐CS@RBP/PAOC‐3@BA/NIR nearly closed by day 2, whereas wounds in the other groups remained visibly open. The wound in the Fe_2_O_3_‐CS/NIR and Fe_2_O_3_‐CS@RBP/NIR groups presented signs of thermal injury, blackening by day 2 and eschar formation by day 5, which were distinct from the regular scabs observed in other groups. Compared to the Fe_2_O_3_‐CS/NIR group, the Fe_2_O_3_‐CS@RBP/NIR group exhibited milder burning scabs, likely due to the targeting effect of RBP, which enabled more precise heat generation at bacterial sites and reduced damage to surrounding healthy tissues. The wound area in the wound region after different treatments was also calculated. Quantitative analysis of wound areas confirmed the superior healing effect of the Fe_2_O_3_‐CS@RBP/PAOC‐3@BA/NIR group, with wound area reduced to only 28% and 7% by day 2 and day 5, respectively. In contrast, wound areas in the control, Fe_2_O_3_‐CS/NIR, Fe_2_O_3_‐CS@RBP/NIR were 91.6% ± 8.5%, 90.6% ± 3.5%, and 79.6% ± 9.0% by day 5, respectively. In addition, the PAOC‐3 (52.3% ± 16.2%) and PAOC‐3@BA (52.3% ± 9.5%) showed significantly smaller wound areas compare to the non‐hydrogel groups: control (PBS/NIR), Fe_2_O_3_‐CS/NIR, and Fe_2_O_3_‐CS@RBP/NIR groups, likely due to the strong adhesive and thermo‐stimulated contraction of PAOC‐3 hydrogel, which mimics the natural wound contraction mechanism, potentially drawing wound edges closer together and facilitating tissue wound closure.

The excellent wound healing performance of Fe_2_O_3_‐CS@RBP/PAOC‐3@BA/NIR treatment could be caused by the synergistic promotion of Fe_2_O_3_‐CS@RBP and PAOC‐3@BA. Upon NIR irradiation, the Fe_2_O_3_‐CS@RBP target *S. aureus* generate localized heat enabling precise photothermal eradication of *S. aureus*. The excess heat generation by Fe_2_O_3_‐CS@RBP transferred to PAOC‐3@BA hydrogel, then PAOC‐3@BA hydrogel undergoes a turbidity transition, forming a light‐scattering barrier that effectively blocking further NIR transmission and stabilizing the temperature near T_cp_ (Figure 5f) to minimizing the risk of thermal damage to healthy tissues. The wound area temperature of mice during the Fe_2_O_3_‐CS@RBP/PAOC‐3@BA/NIR treatment was 47.5 °C (Figure S20, Supporting Information) similar to that in vitro condition ≈47.2 °C (Figure S17, Supporting Information). Meanwhile, the strong tissue adhesive and thermo‐induced self‐contraction of PAOC‐3@BA accelerates wound closure, while the controlled release of BA effectively suppresses inflammation, ultimately contributing to the enhanced wound healing process.

To further evaluate the antibacterial efficacy, mice from each group were sacrificed at specific time point to quantify the bacterial loads in the wound tissue. As shown in Figure 5f, the Fe_2_O_3_‐CS@RBP/NIR and Fe_2_O_3_‐CS@RBP/PAOC‐3@BA/NIR groups exhibited a seven‐log reduction in bacterial counts at both day 2, demonstrating the potent antibacterial activity of Fe_2_O_3_‐CS@RBP under NIR irradiation. Notably, bacterial counts in the Fe_2_O_3_‐CS /NIR group remained two orders of magnitude higher than in the Fe_2_O_3_‐CS@RBP/NIR group, highlighting the enhanced targeting and antibacterial efficiency conferred by RBP functionalization.

To investigate the inflammatory response, concentrations of TNF‐α and IL‐10 were measured in the wounds of mice on day 14 (Figure 5g,h). The Fe_2_O_3_‐CS ‐CS@RBP/PAOC‐3@BA/NIR group showed significantly reduced levels of the pro‐inflammatory cytokine TNF‐α and increased levels of the anti‐inflammatory cytokine IL‐10 compared to other groups, indicating effective inflammation resolution and transition toward the proliferative phase of wound healing.

**Figure 6:**
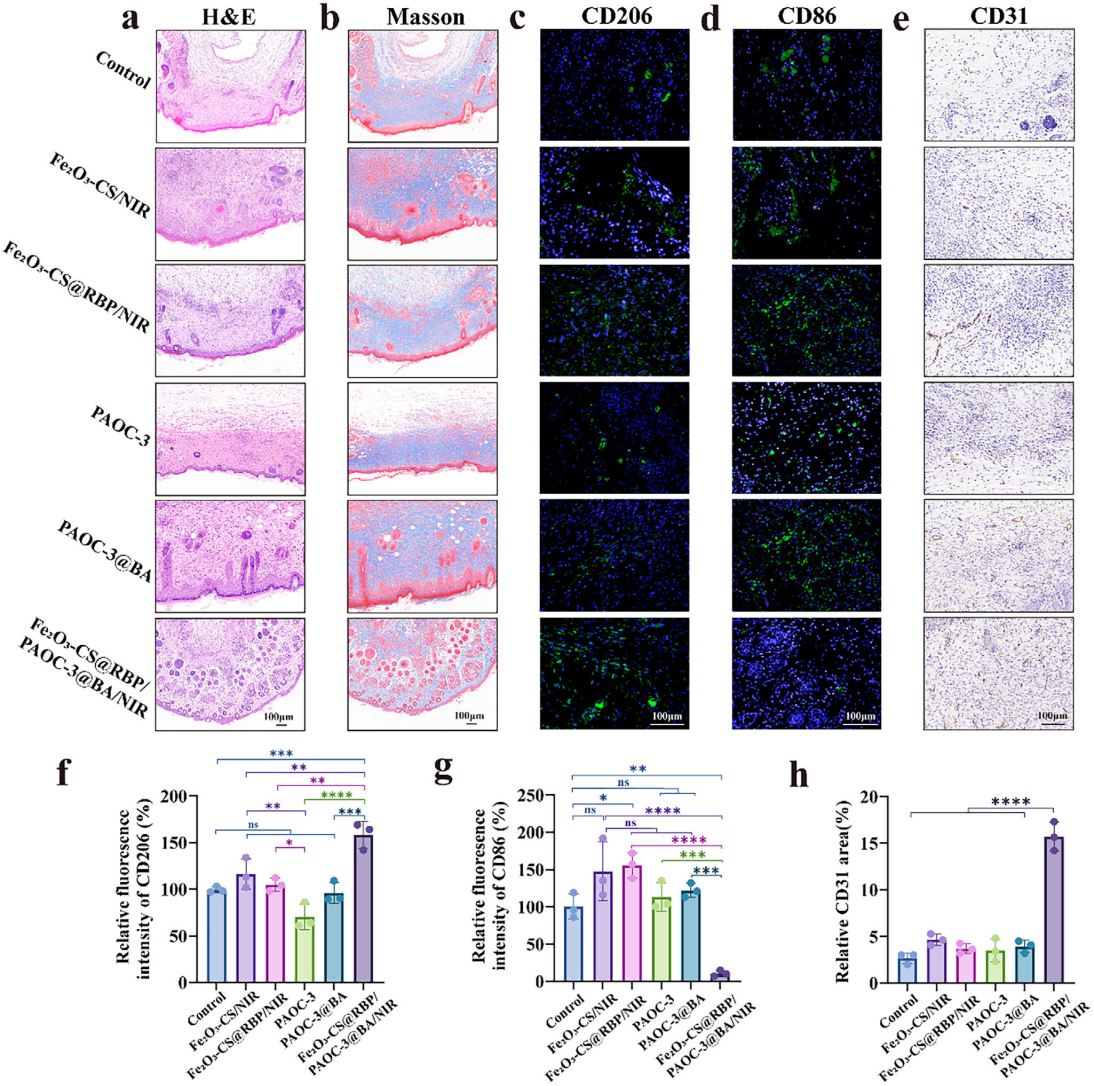
Histological analysis of wound tissue in vivo. a), Representative images of the H&E staining of wound area on day 14 post various treatments. b), Representative images of the Masson staining of wound area on day 14 post various treatments. c,d). Representative images of immunofluorescence staining for CD206 (c) and CD86 (d) on day 14 post various treatments. e), Representative images of immunohistochemical staining for CD31 on day 14 post various treatments. The scale bar is 100 µm. Experiment was repeated three times with similar results. f‐g), Statistical analysis of the relative area of M1 (f) and M2 (g) macrophages. h), Statistical analysis of the relative area of CD31. Data are presented as mean ± standard deviation (n = 3 biological replicates). The statistical significance of the data was assessed using one‐way ANOVA followed by Tukey's multiple comparisons test. *p<0.05, **p<0.01, ***p<0.001, ****p<0.0001.

Histological analysis (HE and Masson staining) was carried out further to evaluate the wound‐healing effect of different treatment by day 14 (**Figure** **6a,b**). The Fe_2_O_3_‐CS@RBP/PAOC‐3@BA/NIR‐treated wounds exhibited minimal inflammatory cell infiltration, reduced epidermal disruption, increased neovascularization and hair follicle regeneration, and well‐organized fibroblast proliferation, indicating robust tissue regeneration, confirming its superior healing effects.

To investigate immune modulation, immunofluorescence staining was performed on wound sections to assess macrophage polarization. After 14 days of treatment, the Fe_2_O_3_‐CS@RBP/PAOC‐3@BA/NIR group showed a marked decrease in M1 macrophages (CD86⁺) and an increase in M2 macrophages (CD206⁺) compared to other groups (Figure 6c–f). Quantitative analysis using ImageJ confirmed significantly lower CD86 expression and higher CD206 expression in this group, suggesting effective suppression of inflammation and promotion of a pro‐healing immune microenvironment.

In addition, immunohistochemical staining for CD31 (a marker of endothelial cells involved in vascular differentiation) and collagen (type I and type III)^[^ ^58^, ^59^ ^]^ was performed to assess angiogenesis and collagen remodeling. As shown in Figure 6g,h, a substantial number of newly formed blood vessels were observed in the Fe_2_O_3_‐CS@RBP/PAOC‐3@BA/NIR group, along with robust collagen deposition. These findings demonstrate that Fe_2_O_3_‐CS@RBP/PAOC‐3@BA/NIR treatment not only facilitates the transition from the inflammatory to the proliferative phase but also significantly accelerates the overall wound healing process.

## 3 Conclusion

In this study, we developed an intelligent, efficient therapeutic system for the enhanced healing of *S. aureus*‐infected wounds. The system integrates a thermo‐responsive hydrogel (PAOC‐3@BA) with NIR‐absorbing, bacteria‐targeting photothermal nanoparticles (Fe_2_O_3_‐CS@RBP), exhibiting potent antibacterial efficacy and accelerating wound healing within one week. Upon NIR irradiation, Fe_2_O_3_‐CS@RBP nanoparticles specifically target *S. aureus* and generate localized heat, enabling precise photothermal eradication of bacteria. The excess heat is transferred to the PAOC‐3@BA hydrogel, which undergoes a turbidity transition to form a light‐scattering barrier. This barrier effectively blocks further NIR transmission, stabilizing the temperature near the T_cp_ and minimizing the risk of thermal damage to surrounding healthy tissues. Simultaneously, the high tissue adhesive and heat‐induced self‐contraction of PAOC‐3@BA promotes rapid wound closure, while the stimuli‐responsive release of the anti‐inflammatory agent BA effectively suppresses inflammation. Together, these effects synergistically contribute to a significantly accelerated and more complete wound healing process. One limitation of this study is that it remains unclear whether the hybrid system would be effective in polymicrobial wound infections involving multiple bacterial species. It would be valuable to construct a polymicrobial infection model, incorporating *S. aureus*, *E. coli*,^[^ ^60^ ^]^ and *P. aeruginosa*,^[^ ^61^ ^]^ to further evaluate the broad‐spectrum antibacterial efficacy and therapeutic potential of the hybrid system in more clinically relevant scenarios. Overall, this work presents a novel and integrated therapeutic strategy that combines self‐regulating photothermal therapy, targeted bacterial recognition, tissue adhesion, heat‐induced self‐contraction, and controlled anti‐inflammatory drug release. This intelligent platform offers significant potential for the safe and effective treatment of *S. aureus*‐induced skin infections, and establishes a promising direction for future clinical applications in infectious wound management.

## 4 Experimental Section

### Ethics Statement

All animal experiments were conducted in accordance with the Guide for the Care and Use of Laboratory Animals issued by the U.S. National Institutes of Health. All experimental protocols involving animals were reviewed and approved by the Animal Research Committee of Sichuan Agricultural University (Approval No. 20240072).

### Materials

ClonExpress Ultra One Step Cloning Kit, 2 × Phanta Max Master Mix (Dye Plus), DL5000 DNA Marker, DL15000 DNA Marker, FastPure Gel DNA Extraction Mini Kit, FastPure Plasmid Mini Kit were purchased from Vazyme Biotech Co., Ltd. (Nanjing, China); Luria–Bertani broth (LB) and Tryptic Soy Broth (TSB) were purchased from Qingdao Hope Bio‐Technology CO., Ltd.; Prestained Protein Marker (10–180 kDa), and 4′, 6diamidino‐2‐phenylindole (DAPI) were purchased from Labgic Bioechnology Co., Ltd. (Beijing, China); Isopropyl‐β‐D‐thiogalactopyranoside (IPTG), Ni‐NTA agarose HP, ColorMixed Protein Marker(11 245 KDa), CCK‐8 Cell Proliferation and Cytotoxicity Assay Kit, Bicinchoninic Acid Protein Assay Kit, Live‐Dead Staining Kit and ECL Western Blotting Substrate were purchased from Beijing Solarbio & Technology Co., Ltd. (Beijing, China); His‐Tag (6*His) Monoclonal antibody and horseradish peroxidase‐conjugated Goat Anti‐Mouse IgG were purchased from Proteintech Group, Inc (Rosemont, USA); FeCl_2_·4H_2_O, sodium acetate (NaOAc), chitosan (CS), Hyaluronic acid (HA) were purchased from Shanghai Macklin Biochemical Co., Ltd. (Shanghai China); Poly(ethylene glycol) (N‐hydroxysuccinimide 5pentanoate) ether N ′ ‐(3‐maleimidopropionyl)aminoethane (MW = 5000 Da, MAL PEG_5000_‐NHS) were purchased from Sigma‐Aldrich (St. Louis, USA); FeCl_3_, Baicalin (BA, Hydroxylammonium chloride, Ethylene glycol, N‐isopropylacrylamide (NIPAM), 2‐acrylamido‐2‐methylpropanesulfonic acid (AMPS), ammonium persulfate (APS), N, N, N′, N′‐tetramethylethylenediamine (TEMED) and periodate solution (NaIO_4_) were purchased from Shanghai Aladdin Biochemical Techology Co., Ltd. (Shanghai China); Rhonox‐1 and Lipopolysaccharides (LPS) were purchased from MedChemExpress Co., Ltd.(Shanghai China) N, N′‐methylene diacrylamide (MBA) was purchased from Chronchem Chemicals Co., Ltd. TNF‐α, IL‐6, IL‐10, IL‐4 ELISA kits were purchased from Mlbio Co., Ltd.(Shanghai China). FITC‐labeled CD86 and FITC‐labeled CD206 were purchased from Proteintech (Wuhan China). Tribromoethanol was purchased from Nanjing AIBI Bio‐Technology Co., Ltd. (Nanjing, China).

### Bacterial Strains and Cells Used

All bacterial strains used in this study are listed in Table S3 (Supporting Information)

### Animals

Six‐week‐old female SPF‐grade ICR mice were obtained from Chengdu Dossy Experimental Animals Co., Ltd. These mice were housed at the Animal Center of the College of Veterinary Medicine, Sichuan Agricultural University, under standard conditions, with free access to food and water at all times. The light cycle was set from 7:30 am to 7:30 pm, while the temperature was controlled at 22 ± 2 °C and the humidity at 40–70%. All animal‐related experimental procedures were performed in compliance with the guidelines of the Animal Care and Use Committee of Sichuan Agricultural University. Animal experiments were independently carried out using different cohorts of mice.

### Expression, Purification, and Characterization of RBP

The RBP gene (Table S1, Supporting Information) and the expression vector pRSF‐Cys‐His_6_‐GFP were amplified using 2× TransStart FastPfu PCR SuperMix (Cat. No. AS221‐02, TransGen Biotech Co., Ltd., Beijing, China). The amplified RBP gene was inserted into the pRSF‐Cys‐His_6_‐GFP vector using the pEASY‐Basic Seamless Cloning and Assembly Kit (Cat. No. CU201‐03, TransGen Biotech Co., Ltd., Beijing, China), resulting in the construction of the pRSF‐Cys‐His_6_‐GFP‐RBP plasmid encoding RBP. The accuracy of the constructed plasmid was confirmed by DNA sequencing (Chengdu Youkangjianxing Biotechnology Co., Ltd., Chengdu, China). Following sequence verification, the pRSF‐Cys‐His_6_‐GFP‐RBP plasmid was transformed into *E. coli* BL21(DE3) competent cells. Transformed colonies were inoculated into 20 mL of LB medium supplemented with 20 µg mL^−1^ kanamycin and cultured overnight at 37 °C with shaking at 220 rpm. The overnight culture was diluted 1:50 into 1 L of TSB medium (also containing 20 µg mL^−1^ kanamycin) and incubated at 37 °C until the OD_600_ reached ≈0.6. The culture was then chilled on ice for 10 min, and protein expression was induced by adding IPTG to a final concentration of 0.5 mM. The induced culture was incubated at 18 °C for 24 h with shaking at 220 rpm. Cells were harvested by centrifugation at 6000 ×g for 15 min, and the resulting pellets were resuspended in lysis buffer (50 mM Tris‐HCl, 2 mM EDTA, 100 mM NaCl, 0.5% Triton X‐100, pH 8.5). Cell disruption was performed by sonication for 20 min, after which the lysate was clarified by centrifugation at 12000 ×g for 20 min. The supernatant was filtered through a 0.45 µm membrane and applied to a Ni‐NTA agarose affinity column (Beijing Solarbio Science & Technology Co., Ltd., Beijing, China) pre‐equilibrated with binding buffer (50 mM NaH_2_PO_4_, 500 mM NaCl, 10 mM imidazole, pH 8.0). The column was washed with 12 column volumes (CV) of wash buffer (50 mM NaH_2_PO_4_, 500 mM NaCl, 20 mM imidazole, pH 8.0) to remove non‐specifically bound proteins. The RBP protein was then eluted with 6 CV of elution buffer (50 mM NaH_2_PO_4_, 500 mM NaCl, 500 mM imidazole, pH 8.0). For further purification, the eluted protein was subjected to size‐exclusion chromatography using a prepacked gel filtration column (HiPrep™ 16/60 Sephacryl® S‐200 HR, GE Healthcare, Cat. No. 17‐1166‐01). The purity of the final protein product was evaluated by 8% SDS‐PAGE followed by Coomassie Brilliant Blue staining. In addition, Western blotting was performed using a 1:20000 dilution of anti‐His (6×His) monoclonal antibody and a 1:5000 dilution of HRP‐conjugated AffiniPure Goat Anti‐Mouse IgG (H+L).

### Synthesis and Characterization of Fe_2_O_3_‐CS and Fe_2_O_3_‐CS@RBP Nanoparticles

Fe_2_O_3_‐CS nanoparticles were synthesized via a hydrothermal method following previously reported protocols,^[^ ^35^, ^36^ ^]^ with slight modifications. Briefly, 0.1 g of FeCl_3_ and 0.05 g of FeCl_2_·4H_2_O were dissolved in 30 mL of distilled water. Then, 0.5 g of sodium acetate (NaOAc) and 36 mg of chitosan (CS) were added to the solution under vigorous stirring for 30 min. The resulting mixture was transferred to a 50 mL Teflon‐lined stainless‐steel autoclave and maintained at 120 °C for 8 h. After cooling to room temperature, the precipitate was collected by centrifugation, washed three times with ethanol, and dried at 60 °C for 3 h. To prepare Fe_2_O_3_‐CS@RBP, 10 mg of Fe_2_O_3_‐CS was resuspended in 1 mL of ultrapure water and incubated with 0.2 mg of MAL‐PEG_5000_‐NHS under gentle stirring at room temperature for 1 h to activate surface maleimide groups. The mixture was then centrifuged at 8000 ×g for 10 min to remove unreacted components, yielding maleimide‐functionalized Fe_2_O_3_‐CS. Subsequently, 400 µL of RBP solution (5 mg mL^−1^) was added dropwise to the Fe_2_O_3_‐CS suspension and stirred at room temperature for 12 h. The RBP protein, engineered with a free N‐terminal cysteine residue, covalently conjugated to the maleimide‐functionalized Fe_2_O_3_‐CS via a thiol‐maleimide reaction, generating Fe_2_O_3_‐CS@RBP. The morphology of Fe_2_O_3_‐CS and Fe_2_O_3_‐CS@RBP was observed using transmission electron microscopy (TEM, Talos F200E, Thermo Fisher Scientific, USA). Successful RBP conjugation was further confirmed by confocal laser scanning microscopy (CLSM), utilizing Rhonox‐1 (yellow fluorescence) to label the Fe of Fe_2_O_3_‐CS@RBP. The hydrodynamic diameter and zeta potential of the nanoparticles in ultrapure water were measured using a Zetasizer Nano ZSE (Malvern Instruments, UK). Thermal stability was evaluated using thermogravimetric analysis (TGA, SDT650, TA Instruments, USA) under a nitrogen atmosphere from room temperature to 1000 °C. Fourier‐transform infrared spectroscopy (FTIR, Spectrum Two, PerkinElmer, USA) was employed to identify characteristic functional groups. UV‐vis absorption spectra were recorded using a UV‐6100 spectrophotometer (MAPADA, China). Finally, X‐ray diffraction (XRD) patterns were obtained with a Malvern Panalytical diffractometer using Cu Kα radiation (λ  =  1.5406 Å) to analyze the crystalline structures of Fe_2_O_3_‐CS and Fe_2_O_3_‐CS@RBP. Inductively coupled plasma mass spectrometry (ICP‐MS) was used to quantify the iron release from nanoparticles Fe_2_O_3_‐CS and Fe_2_O_3_‐CS@RBP after incubation with medium for 1 week.

### *S. aureus*‐Targeting Performance of RBP and Fe_2_O_3_‐CS@RBP

To assess the targeting specificity of the RBP protein and *Fe_2_O_3_*‐CS@RBP nanoparticles toward *S. aureus*, methicillin‐resistant *S. aureus* (MRSA, ATCC 43300) was cultured and diluted to an OD_600_ of 0.2. The bacterial suspension was then incubated with 1 mg mL^−1^ DAPI at 37 °C for 2 h for nuclear staining. Following staining, 400 µL of the DAPI‐treated bacterial suspension was separately incubated with either 100 µL of RBP solution (100 µg mL^−1^) or 10 µL of Rhonox‐1‐labeled Fe_2_O_3_‐CS@RBP suspension (10 mg mL^−1^). The mixtures were incubated at room temperature for 30 min to allow for binding interactions. After incubation, samples were centrifuged to discard the supernatant and remove unbound proteins or nanoparticles. The bacterial pellets were washed three times with PBS and resuspended in 40 µL of PBS. Then, 1 µL of each resuspension was placed onto a 1.5% agarose gel pad for imaging. The binding interaction and targeting efficiency of RBP and Fe_2_O_3_‐CS@RBP with *S. aureus* were observed using confocal laser scanning microscopy (CLSM; Leica Microsystems, Germany).

### Photothermal Performance of Fe_2_O_3_‐CS and Fe_2_O_3_‐CS@RBP

To investigate the photothermal properties of the prepared nanoparticles, suspensions of Fe_2_O_3_‐CS and Fe_2_O_3_‐CS@RBP at various concentrations (1.0, 0.5, 0.25, 0.125, and 0.0625 mg mL^−1^) were prepared. For each concentration, 200 µL of the suspension was transferred into individual containers. An 808 nm near‐infrared (NIR) laser (1.0 W cm^−2^) was applied for 10 min. The temperature rise during irradiation was recorded every 30 seconds using a FLIR thermal imaging camera, and deionized water was used as a negative control. All experiments were conducted at a room temperature of ≈25 °C and repeated in triplicate to ensure reproducibility. To assess photothermal stability, 1 mL of Fe_2_O_3_‐CS@RBP solution (0.5 mg mL^−1^) was subjected to five on/off irradiation cycles, each consisting of 10 min of laser irradiation followed by 10 min of natural cooling. Temperature changes were monitored throughout the cycles using the same thermal camera setup. The photothermal conversion efficiencies (η) of Fe_2_O_3_‐CS and Fe_2_O_3_‐CS@RBP nanoparticles were calculated based on a previously reported method.^[^ ^62^ ^]^ The following equations were used for the calculation:

(1) $$\eta =\frac{hS\left(T_{max}-T_{surr}\right)-Q_{dis}}{I\left(1-10^{-A_{808}}\right)}$$

(2) $$Q_{dis}=hS\left(T_{max,H_{2}O}-T_{surr}\right)$$

(3) $$\tau_{s}=\frac{m_{D}C_{D}}{hS}$$

(4) $$t=-\tau_{s}ln\theta$$

(5) $$\theta =\frac{T_{surr}-T}{T_{surr}-T_{max}}$$

where *h* represents the heat transfer coefficient, *S* represents the surface area of the container, T_max_ represents the maximum temperature of the material solution, T_surr_ represents the ambient temperature. Q_dis_ represents the heat generated by water after absorbing light, 𝑚_𝐷_ is the mass of water, c_𝐷_ is the specific heat capacity of water. τ_𝑠_ is the fitted time constant, and I is the laser power density. A_808_ represents the absorption value of nanomaterials at 808 nm. *t* represents the cooling time, T is the temperature at t seconds of cooling, and *θ* is a dimensionless dynamic temperature introduced in the calculation of τ_𝑠_.

### Synthesis of OHA

The sodium periodate solution (NaIO_4_ 0.1 g mL^−1^) was slowly added to the hyaluronic acid (HA,0.01 g mL^−1^) solution under continuous stirring and allowed to react for 12 h in the dark at room temperature. After the oxidation reaction, 1 mL of ethylene glycol was added to quench the excess sodium periodate. The reaction mixture was subjected to dialysis (molecular weight cut‐off: 14000 Da) against ultrapure water for 3 days. The oxidation degree of OHA was determined using a hydroxylamine hydrochloride‐based titration method as previously described in the literature.^[^ ^62^ ^]^ Briefly, 0.1 g of OHA was dissolved in 25 mL of ultrapure water, and 0.4343 g of hydroxylamine hydrochloride was added. The reaction mixture was stirred in the dark at room temperature for 24 h. Following the reaction, 100 µL of 0.05 M NaOH standard solution was added stepwise to the system, with stirring for 10 seconds after each addition. The pH was recorded after each addition. The increase in pH was monitored, and the titration was terminated when the pH increment began to decrease or returned to its initial level. The oxidation degree (OD%) of OHA was calculated using the following Equation (6):

(6) $$Oxidation\;degree=\frac{\Delta V\times c\times 402\times 0.001}{2w}\times 100\%$$

Here, ΔV represents the volume (in mL) of the NaOH standard solution consumed when the pH increment is at its maximum; c is the molar concentration (mol/L) of the NaOH standard solution; 402 is the molecular weight (g/mol) of the OHA repeating unit; and w is the mass (g) of OHA.

### Synthesis and Characterization of PAOC and PAOC‐3@BA hydrogels

Hydrogels with varying formulations were synthesized via free radical polymerization.^[^ ^62^ ^]^ Initially, 200 mg of N‐isopropylacrylamide (NIPAM) was dissolved in 2 mL of ultrapure water under continuous magnetic stirring. Subsequently, 10 mg of 2‐acrylamido‐2‐methylpropanesulfonic acid (AMPS) and 6 mg of N,N′‐methylenebisacrylamide (MBA) were added as functional monomer and crosslinker, respectively. The mixture was stirred thoroughly for 1 h to ensure complete dissolution and homogeneity. Next, 1 mg of OHA and varying amounts of chitooligosaccharide (COS) (0.1 mg, 0.5 mg, and 1.0 mg) were added sequentially to the solution under stirring. The formulations were designated as follows based on the COS content: PAOC (0 mg COS), PAOC‐1 (0.1 mg COS), PAOC‐2: (0.5 mg COS), and PAOC‐3: (1.0 mg COS). After an additional 1 h of stirring, ammonium persulfate (APS) was added to initiate the cross‐linking reaction. The precursor solution was stirred for another 1 h, after which 5 µL of N,N,N′,N′‐tetramethylethylenediamine (TEMED) was added to accelerate gelation. The solution was then left to stand at room temperature until formation of the hydrogel was complete. To prepare the PAOC@BA hydrogel, 5 mg of baicalin (BA) powder was dissolved in 500 µL of PBS by vigorous shaking to ensure complete dissolution. Then, 200 µL of the BA solution and 5 µL of TEMED were added to the hydrogel precursor solution and mixed thoroughly using a magnetic stirrer to form a uniform mixture. The resulting solution was quickly dispensed into molds and allowed to stand at 4 °C for 2 h to complete gelation. FTIR (PerkinElmer, USA) and ^13^C NMR spectrometer (Bruker Advance III, Germany) was employed to identify characteristic functional groups of hydrogels. SEM (VEGA3, TESCAN,Czech) was used to observe the morphology of different hydrogels.

### Sweling Ratio (SR) Measurement

The sweling behavior of each hydrogel formulation was evaluated by the gravimetric (weighing) method to determine the swelling ratio (SR) over time in distilled water. Briefly, the initial dry weight of each hydrogel sample was recorded as 𝑊𝑖 using an electronic balance. The samples were then immersed in ultrapure water at room temperature, and allowed to swell to equilibrium. At predetermined time intervals, the samples were removed, gently blotted with filter paper to remove surface water, and immediately weighed to obtain the swollen weight 𝑊𝑡. The swelling ratio (SR) was calculated using the following Equation (7):

(7) SR=(Wt−Wi)/Wi×100%

### Rheological Properties Test

The rheological properties of the hydrogel formulations were evaluated to assess their mechanical strength using a rheometer (MCR92, Anton Paar). For the dynamic rheological measurements, hydrogel samples with a diameter of 25 mm and a thickness of 2 mm were prepared and placed between two parallel plates (40 mm in diameter), with a fixed gap of 2 mm. The storage modulus (G′) and loss modulus (G″) were measured as functions of angular frequency in the range of 0.1–100 rad s^−1^, under a constant shear strain amplitude of 1%, which was within the linear viscoelastic region. All measurements were performed at 25, 37, and 48 °C, respectively.

### Tissue Adhesion of Hydrogels

The tissue adhesion properties of each hydrogel were evaluated using an improved tensile test method with a universal testing machine (TY8000‐A, Tian Yuan Test Instrument).Two pieces of fresh pigskin were fixed to the upper and lower clamps of the machine at a controlled temperature of 37 °C. Hydrogels with uniform dimensions (16 mm diameter, 4 mm thickness) were applied between the facing surfaces of the pigskins to simulate tissue bonding.

The maximum tensile force required to separate the two pigskins was recorded. The adhesive strength was calculated based on the peak force prior to complete detachment.

### Transmittance at Different Temperatures

The temperature‐responsive optical transmittance of hydrogels was measured at 808 nm using a UV–Vis–NIR spectrophotometer (UV‐2600, Shimadzu, Japan). Briefly, Hydrogel samples with thicknesses of 2, 4, 8, and 10 mm were gradually heated from room temperature to 55 °C at a controlled heating rate of 2 °C per 10 min. Transmittance values were recorded at 2 °C intervals to monitor the thermos‐responsive behavior of the hydrogels.

### Thermostatic Control of the Photothermal Nanoparticle–Hydrogel Composite System

To evaluate the thermostatic behavior of the hydrogel–nanoparticle composite system, a suspension of Fe_2_O_3_‐CS@RBP nanoparticles (0.5 mg mL^−1^) was prepared and transferred into a transparent container. After the container was filled with the nanoparticle suspension, a pre‐cut hydrogel sample was carefully placed on top of the suspension, forming a sealed interface. The entire system was then subjected to 808 nm near‐infrared (NIR) laser irradiation for 10 min, with the laser beam directed perpendicularly to the hydrogel surface. The hydrogel types tested included PAOC@BA (4 mm in thickness), PAOC‐1@BA (4 mm in thickness), PAOC‐2@BA (4 mm in thickness), and PAOC‐3@BA (4 mm in thickness), each in combination with Fe_2_O_3_‐CS@RBP nanoparticles. Temperature changes within the system were monitored in real‐time using an infrared thermal imaging camera, allowing for both quantitative temperature mapping and visual observation of thermal effects. Simultaneously, turbidity changes in the hydrogels were recorded photographically to assess their phase transition behavior under photothermal activation.

### Biocompatibility Evaluation

Vero cells, HepG2 cells, and HEK‐293T cells were used to evaluate the cytocompatibility of hydrogel extracts and Fe_2_O_3_‐based nanoparticles through a CCK‐8 cell viability assay. Hydrogel samples (14 mm in diameter,4 mm in thickness) were then cocultured with Vero cells for 24 h. Separately, Fe_2_O_3_‐CS and Fe_2_O_3_‐CS@RBP nanoparticles were tested at concentrations of 0.0625, 0.125, 0.25, 1.0, and 2.0 mg mL^−1^, and cocultured with Vero cells for 24 h to evaluate dose‐dependent cytotoxicity. Following incubation, 100 µL of DMEM containing 10% Cell Counting Kit‐8 (CCK‐8) reagent (Dojindo Molecular Technologies, Japan) was added to each well and incubated at 37 °C for 1 h. The absorbance at 450 nm was measured to assess cell viability.

The hemocompatibility of hydrogel samples and nanoparticles was assessed using a standard hemolysis assay with rabbit erythrocytes. Fresh blood was collected from healthy rabbits and centrifuged at 116 × g for 10 min to isolate erythrocytes. The red blood cells (RBCs) were washed three times with sterile phosphate‐buffered saline (PBS) and then diluted to a final concentration of 2% (v/v) in PBS. PAOC, PAOC‐3@BA (14 mm in diameter, 4 mm in thickness), Fe_2_O_3_‐CS (2 mg mL^−1^), and Fe_2_O_3_‐CS@RBP (2 mg mL^−1^), were added to PBS containing 2% (v/v) erythrocytes. The cells were incubated at 37 °C for 1 h and then centrifuged for 15 min at 3,000 g. The supernatant was transferred to a 96‐well plate, and the absorbance at a wavelength of 570 nm was measured with a microplate reader. The absorbance relative to the positive control, which was treated with 10% Triton X‐100, was defined as the percentage of hemolysis.

### Histological Analysis

To assess the in vivo biocompatibility of the hydrogel–nanoparticle composite system, a histopathological examination was conducted on major organs from *S. aureus*‐infected wound mice following 48 h of treatment. After euthanization, the heart, liver, spleen, lung, and kidney were harvested, fixed in 4.0% paraformaldehyde, and subsequently embedded in paraffin. Tissue sections were cut at a thickness of 5 µm and stained using hematoxylin and eosin (H&E). Histopathological changes were observed using a pathology slide scanner (Olympus, VS120‐S6‐W, Japan). For each organ type, three sections from different regions were analyzed in three mice per group. To evaluate in vivo wound healing, mice were sacrificed on day 14 post‐treatment, and tissues from the wound site along with the heart, liver, spleen, lung, and kidney were collected. All samples were fixed in 4% paraformaldehyde, embedded in paraffin, and sectioned at 5 µm thickness. The sections were embedded in paraffin and subjected to H&E, Masson, immunohistochemical, and immunofluorescence staining.

### Antibacterial Activity of NPs and Hydrogel–Nanoparticle Composite System

Methicillin‐resistant *S. aureus* (MRSA) was selected to assess the antibacterial efficacy of photothermal nanoparticles and the thermoregulatory control system in vitro. Initially, MRSA was cultured in LB broth at 37 °C and 180 rpm for 18 h. The bacterial cell density was then adjusted to 10^7^ CFU mL^−1^ using PBS. In the nanoparticle antibacterial assay, MRSA at a specific concentration was treated with Fe_2_O_3_‐CS and Fe_2_O_3_‐CS@RBP at concentrations of 0.0625, 0.125, and 0.25 mg mL^−1^, followed by a 10‐min exposure to NIR (808 nm, 1 W cm^−2^), while control groups received no irradiation. For the antibacterial evaluation of the hydrogel‐nanoparticle composite system, Fe_2_O_3_‐CS@RBP concentrations of 0.25, 0.5, and 1 mg mL^−1^ were combined with the bacterial suspension in a transparent container, covered by a coverslip and layer of PAOC‐3@BA hydrogel to create a sealed interface. These samples were subjected to a 10‐min NIR (808 nm, 1 W cm^−2^) or left untreated as a control. Following various treatments, the samples were then placed in a 37 °C incubator for 3 h. Subsequently, the samples were serially diluted 1000 times with PBS, and 10 µL of each diluted bacterial suspension was plated on LB solid medium. The plates were incubated at 37 °C for 18 h to allow colony growth for assessment of antibacterial viability.

### Macrophage Polarization

Murine macrophages RAW264.7 (ATCC) were cultured in DMEM medium supplemented with 10% FBS and 1% penicillin‐streptomycin, then seeded in 6‐well plates for a 24‐h incubation at 37 °C with 5% CO_2_. To replicate an in vitro inflammatory milieu, RAW264.7 cells were exposed to LPS (100 ng mL^−1^) for 12 h to induce M1 polarization. The leaching solution was prepared by incubating PAOC and PAOC@BA hydrogels at 37 °C for 24 h. The thermo‐responsive shrinkage of the hydrogels under these conditions induced the release of leachates into the surrounding medium Subsequently, the leachates was introduced to the polarized M1 macrophages and incubated for 24 h. Following this, the cells were harvested, and the levels of pro‐inflammatory factors (TNF‐α, IL‐6) and anti‐inflammatory factors (IL‐10, IL‐4) were quantified using ELISA. The same protocol was repeated, with CD86 and CD206 antibodies utilized for labeling, and subsequent analysis performed using a flow cytometer (BriCyte E6, Mindray).

### Evaluation of Wound Healing on Mice Model

Six‐week‐old female (SPF) mice were procured from Chengdu Dossy Experimental Animals Co., Ltd. Following a one‐week acclimatization period, the dorsal fur of all mice was shaved. Anesthesia was induced via intraperitoneal injection of trichloroethanol, and a circular full‐thickness wound with a 6 mm diameter was meticulously created on the mice's backs using a sterile wound punch. Subsequently, a MRSA suspension (20 µL, 1 × 10^9^ CFU mL) was gently inoculated onto the wound. After 24 h, the mice were divided into 6 groups (n = 10): Control (without treatment), Fe_2_O_3_‐CS/NIR (treated with 50 µL of 0.5 mg mL^−1^ Fe_2_O_3_‐CS, followed by 10 min of NIR irradiation), Fe2O3‐CS@RBP/NIR (treated with 50 µL of 0.5 mg mL^−1^ Fe_2_O_3_‐CS@RBP, followed by 10 min of NIR irradiation), PAOC‐3 (treated with PAOC‐3 hydrogel only), PAOC‐3@BA (treated with PAOC‐3@BA hydrogel only), and Fe_2_O_3_‐CS@RBP/PAOC‐3@BA/NIR (treated with 50 µL of 0.5 mg mL^−1^ Fe2O3‐CS@RBP, then covered with PAOC‐3@BA hydrogel, followed by 10 min of NIR irradiation). In the NIR treatment groups, the wounds were exposed to NIR (1 Wcm^−2^) for 10 min. After the procedure, the mice were individually caged with free movement and food access. Daily recordings of the mice's body weights were conducted, and the wound sizes were measured using a ruler. On the 2^nd^ and 14th post‐treatment days, 3 mice were randomly selected from each group for sacrifice, and wound tissues were harvested for bacterial quantification. On day 14, TNF‐α and IL‐10 levels of wound tissues homogenate supernatants were assessed using ELISA kits. Throughout the period from day ‐1 to 14, wound photographs were captured using a digital camera. Image J software was employed to determine the wound area, and the wound healing rate was calculated using the Equation (8):

(8) Woundhealingrate(%)=(A0−At)/A0×100%

where A_0_ and A_t_ represented the wound area on day 0 and day t, respectively.

### Statistical Analysis

All statistical analyses were conducted with GraphPad Prism 8 software (GraphPad Software). Experimental data were presented as means ± standard deviation, derived from a minimum of three independent experiments. The statistical significance of the data was evaluated via one‐way ANOVA, followed by Tukey's multiple comparisons test, using GraphPad Prism 8.0. The notations used were as follows: ns, no significance; *p < 0.05; **p < 0.01; ***p < 0.001; ****p < 0.0001.

## Conflict of Interest

The authors declare no conflict of interest.

## Supporting information



Supporting Information

## Data Availability

The data that support the findings of this study are available from the corresponding author upon reasonable request.;
