# Supplementary material for: SMart Nanoparticle–Hydrogel Hybrid System for Synergistic Eradication of Infection and Promotion of Wound Healing
Source: Adv Sci (Weinh). 2025 Nov 30;13(9):e17320. doi: 10.1002/advs.202517320 (PMC12903975; doi:10.1002/advs.202517320)
Supplement: Supplementary file 1 — Supporting Information [file ADVS-13-e17320-s001.docx]

**Supporting Information**

Smart Nanoparticle–Hydrogel Hybrid System for Synergistic Eradication of Infection and Promotion of Wound Healing

Hongping Wan ^a, 1, *^, Huirong Tan ^a, 1^, Xinghong Zhao ^a, b, *^

^a^ Center for Infectious Diseases Control (CIDC), Sichuan Agricultural University, Chengdu, 611130, China.

^b^ State Key Laboratory of Veterinary Public Health and Safety, College of Veterinary Medicine, China Agricultural University, Beijing 100193, China

^1^ Contribute equally and share the first author.

^*^ Correspondence: [hpwan@sicau.edu.cn](mailto:hpwan@sicau.edu.cn) (H. Wan); [x.zhao@cau.edu.cn](mailto:x.zhao@cau.edu.cn) (X. Zhao)


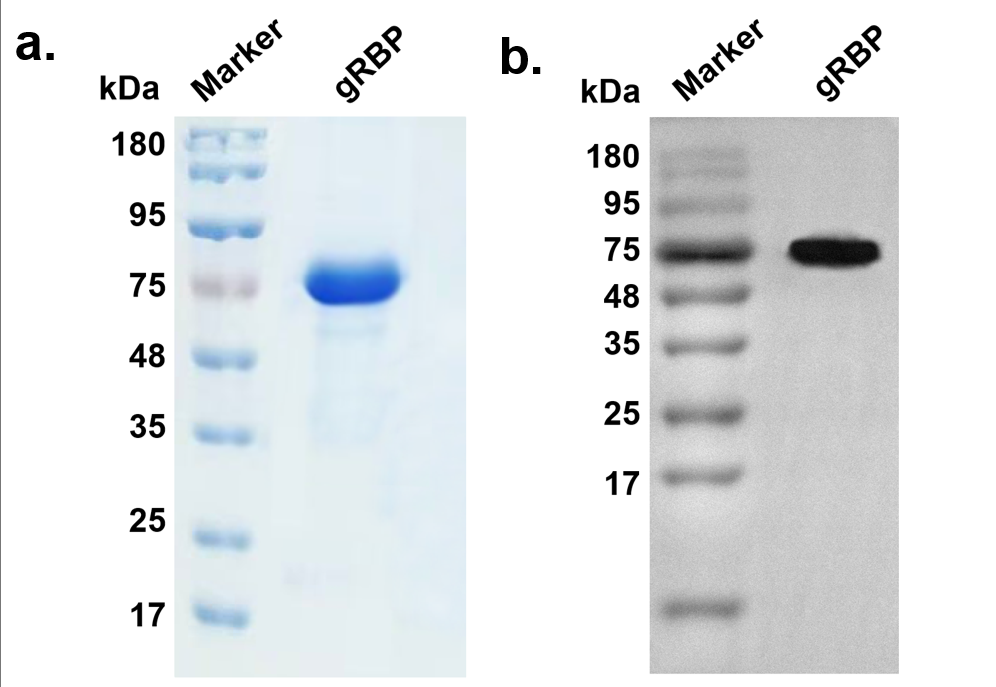


**Figure S1.** a) SDS-PAGE images and b) anti-His_6_ western blot of the heterologously expressed RBP. Experiments were repeated three times with similar results.


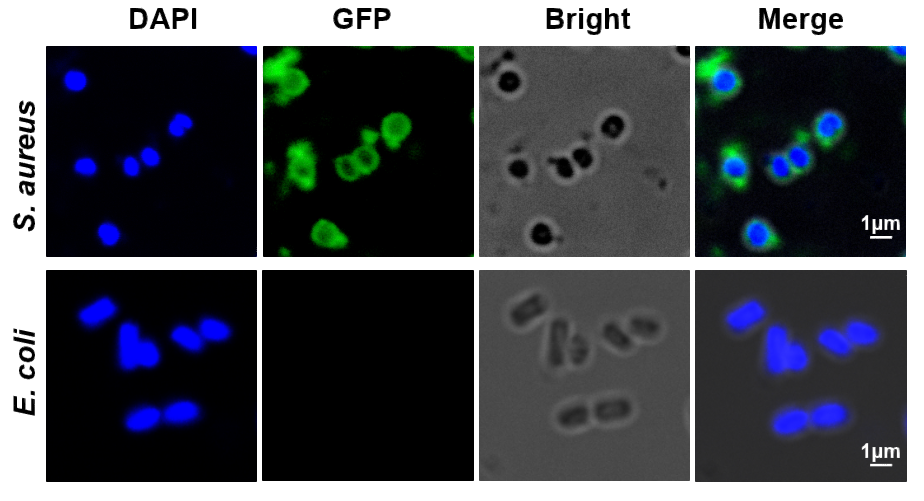


**Figure S2.** Confocal laser scanning microscopy images of MRSA and *E. coli* after incubation with RBP (green) and bacterial nucleoids are stained with DAPI (blue). Experiments were repeated three times with similar results.


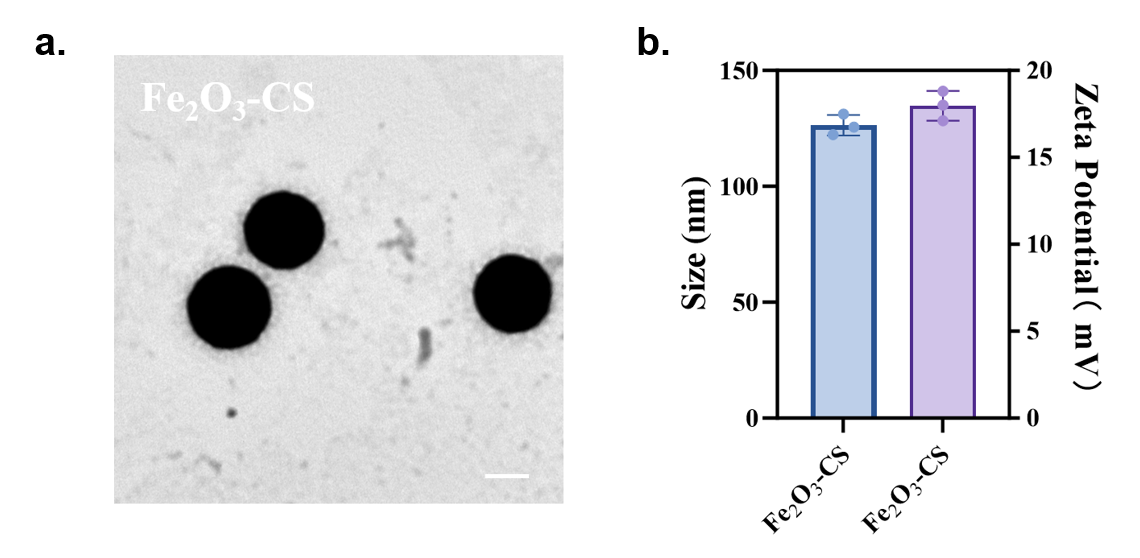


**Figure S3.** a) TEM images (Scale bar: 100 nm). b) Hydrodynamic size and Zeta potential of Fe_2_O_3_-CS in deionized water.


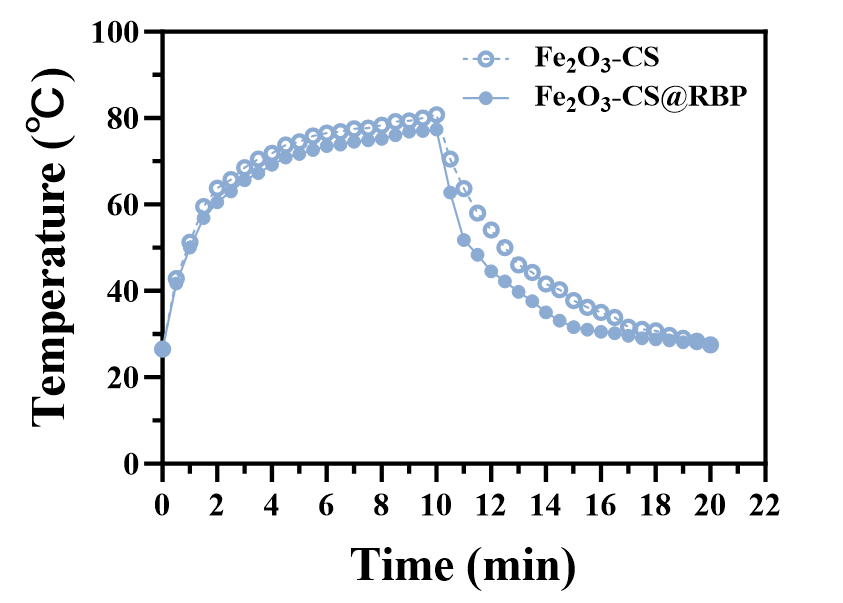


**Figure S4.** Temperature variation of Fe_2_O_3_-CS and Fe_2_O_3_-CS@RBP nanoparticles irradiated by an 808 nm laser (1 W cm^-2^) for 10 min, followed by natural cooling.


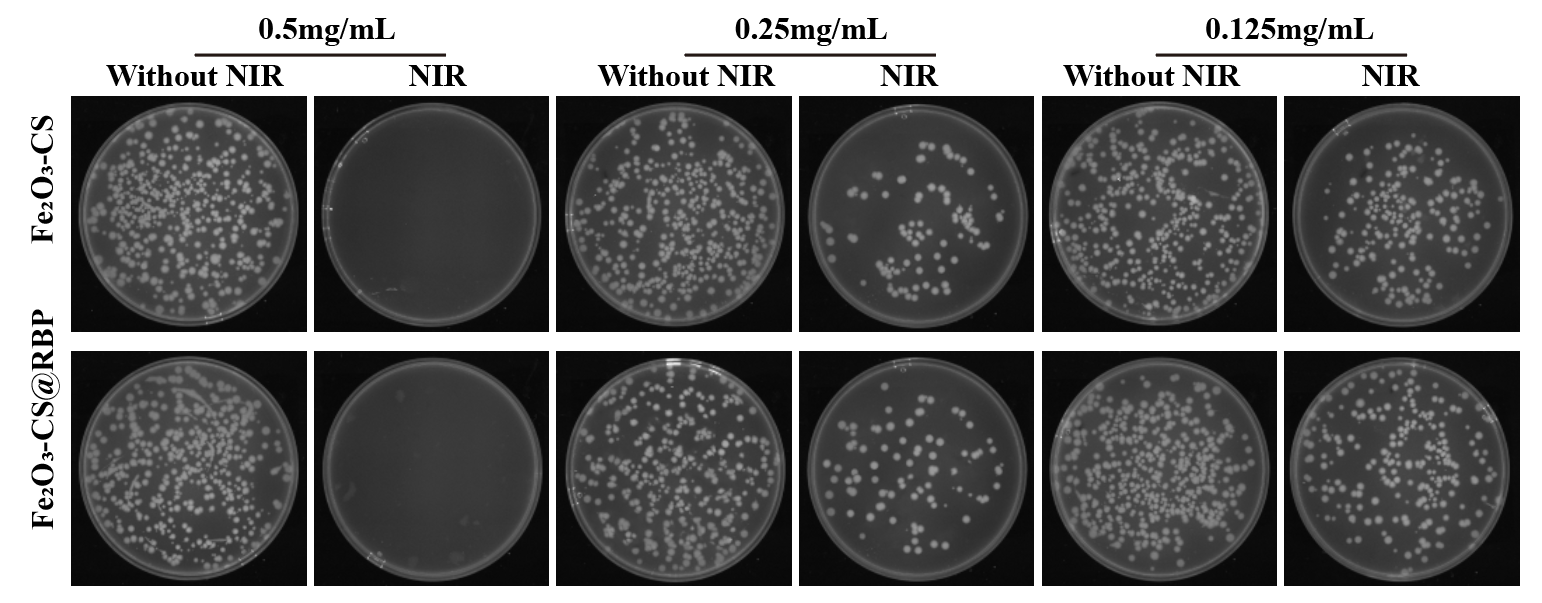

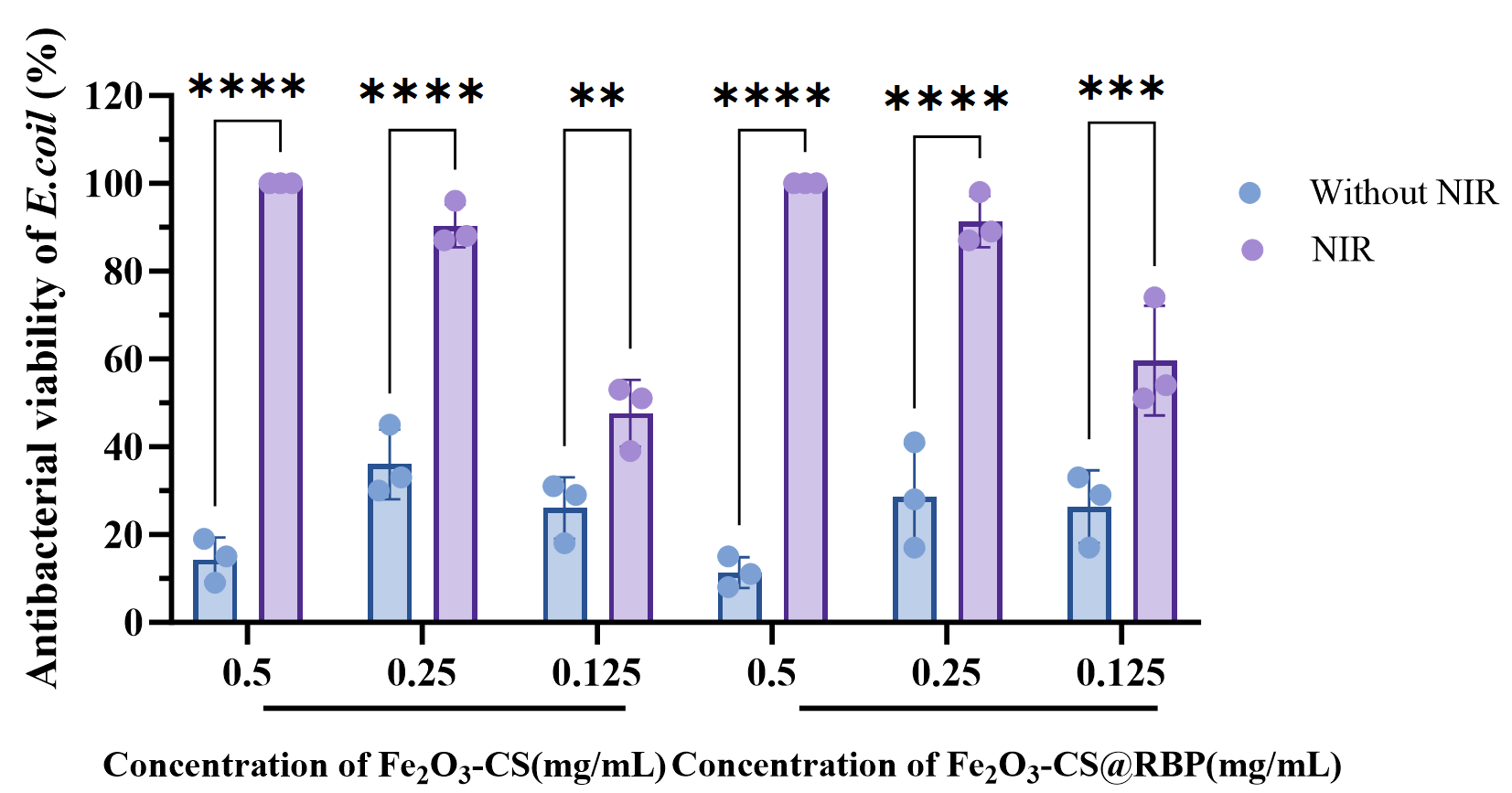


**Figure S5**. Photothermal antibacterial activity of Fe_2_O_3_-CS@RBP and Fe_2_O_3_-CS with different concentration against *E. coli*. Under NIR irradiation, complete bacterial eradication was accomplished in both the Fe_2_O_3_-CS@RBP and Fe_2_O_3_-CS groups at a concentration of 0.5 mg/mL.


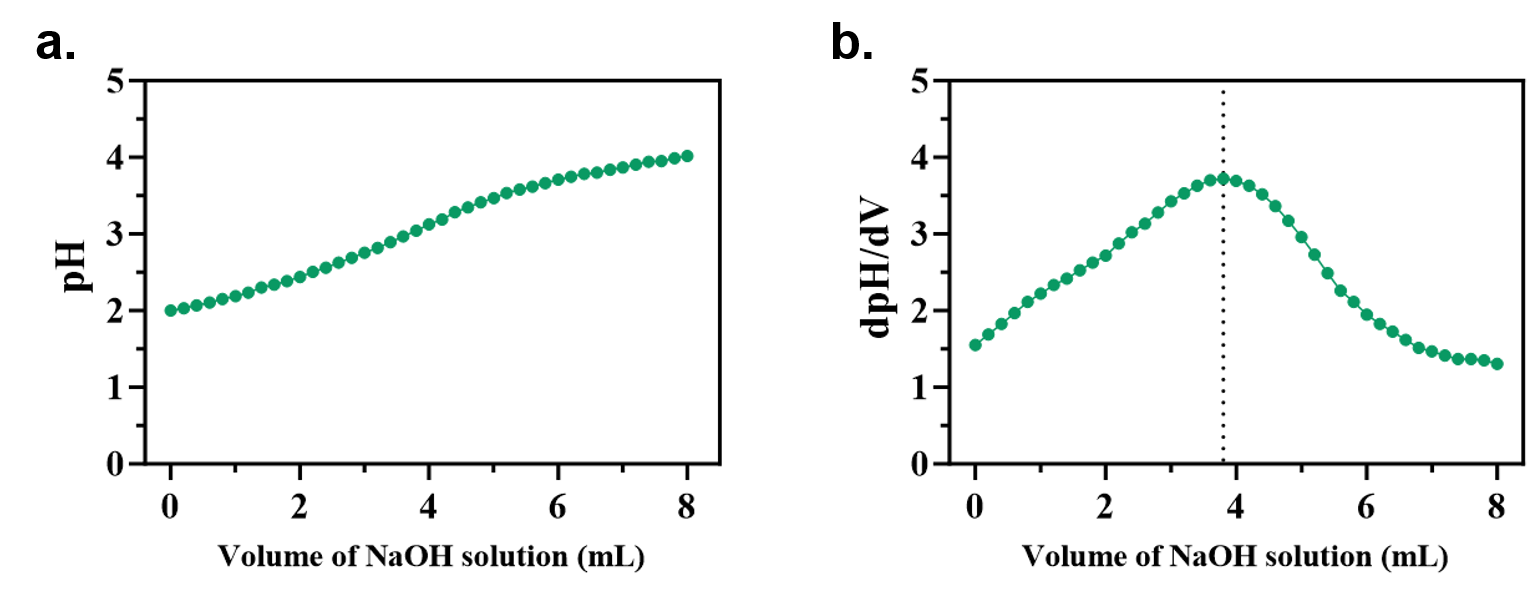


**Figure S6.** a) Curve of pH value varying with the titration volume of NaOH solution. b) First derivative titration curve with the titration volume of NaOH solution.


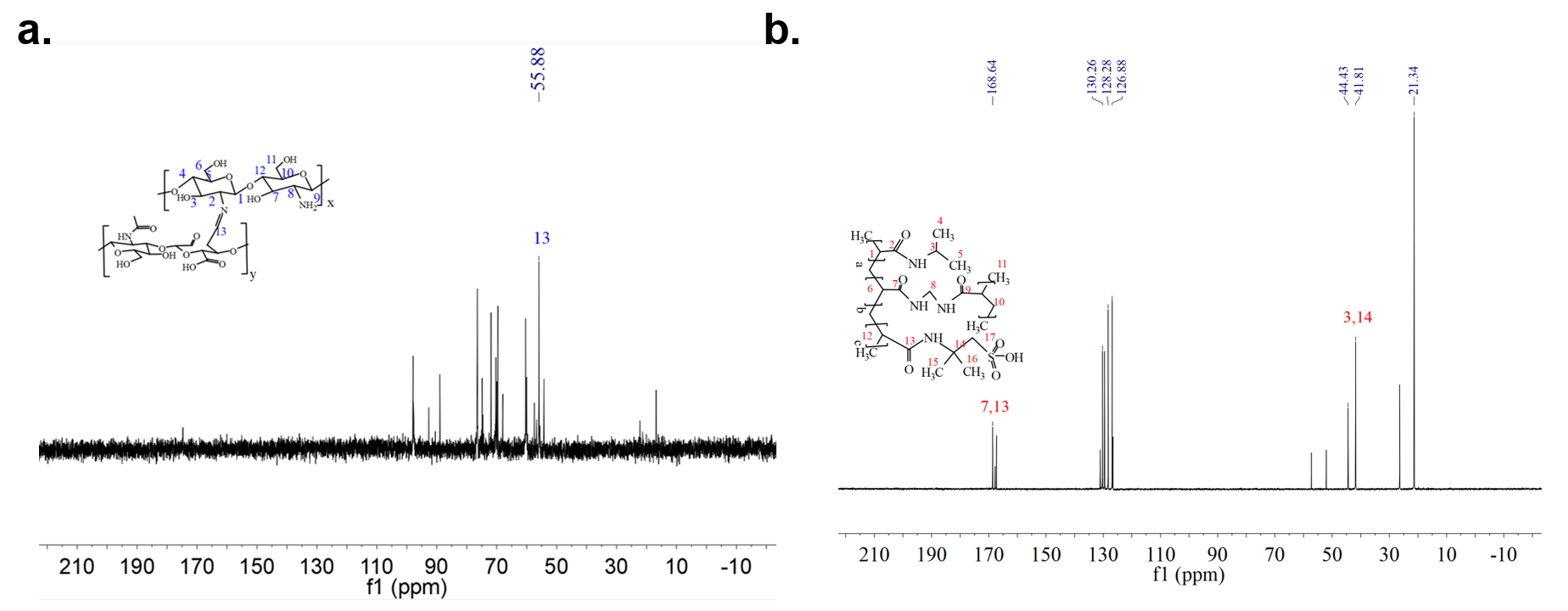


**Figure S7.** a,b) Solid-state ^13^C NMR spectrum of OVA-COS (a) and PNI-AMPS (b) hydrogel.


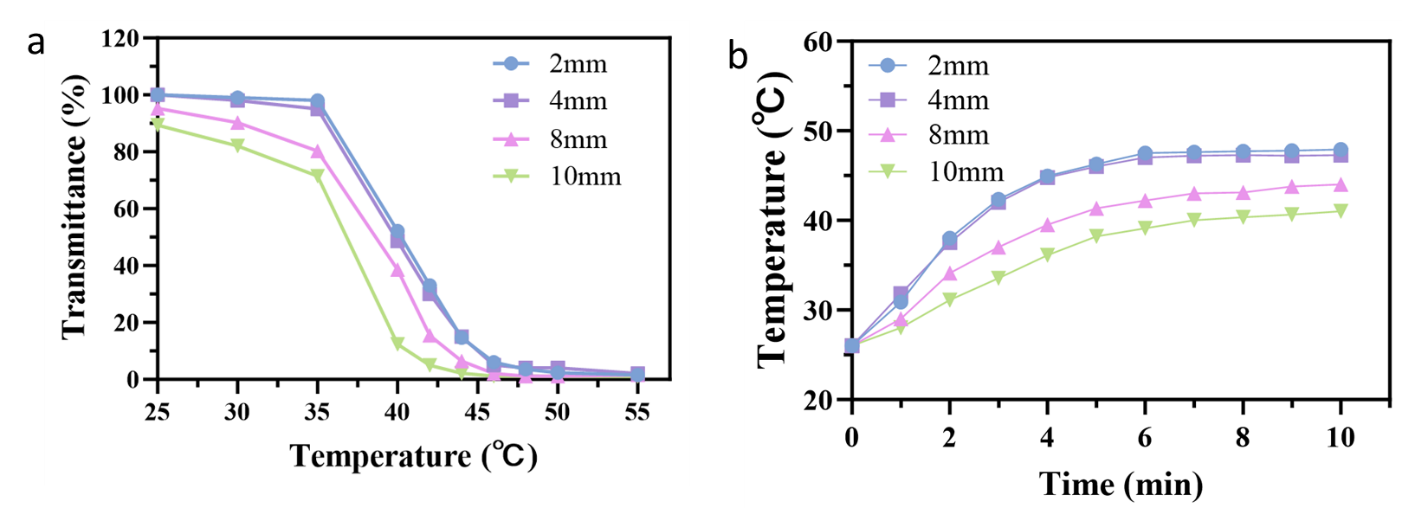


**Figure S8. a)** Transmittance of the PAOC-3@BA hydrogel with different thickness at 808 nm as a function of temperature. b) Photothermal curves of hydrogels with different thickness after being irradiated by 808 nm NIR (1 W cm^-2^) with Fe_2_O_3_-CS@RBP (0.5mg/ml) incorporated.


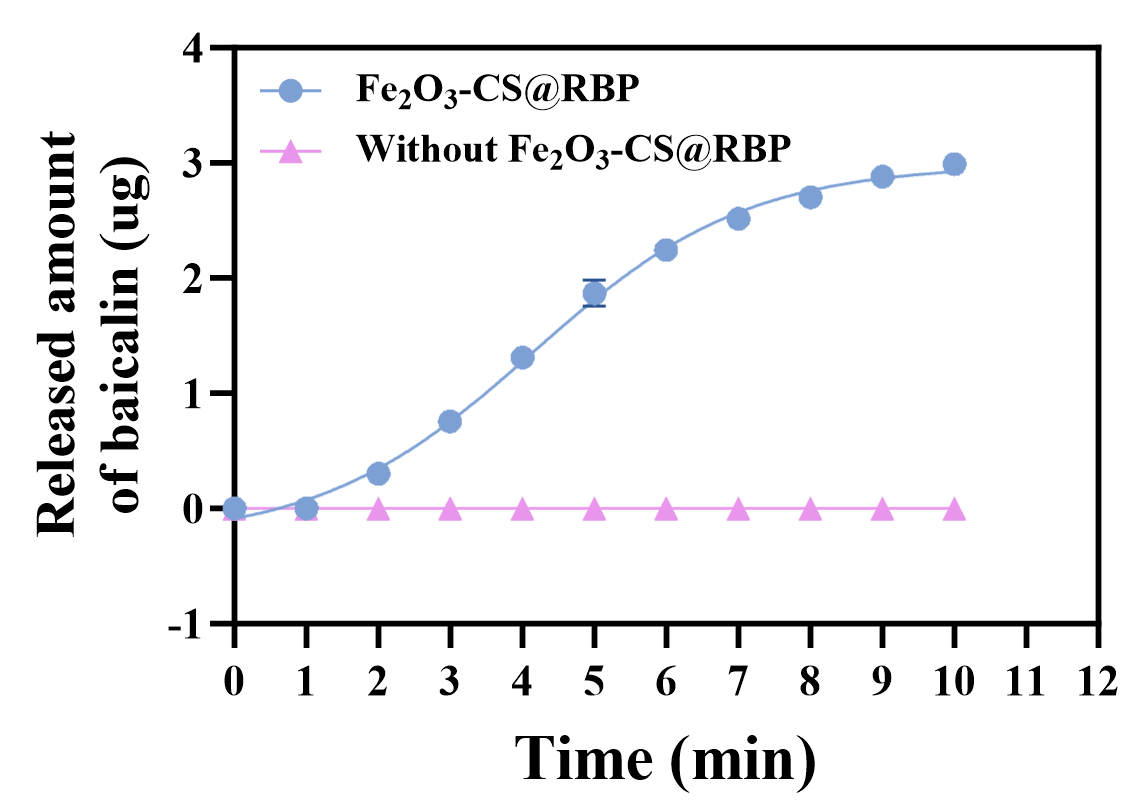


**Figure S9.** The release of BA with or without Fe_2_O_3_-CS@RBP treatment underNIR irradiation (1 W cm^-2^, 10 minutes).


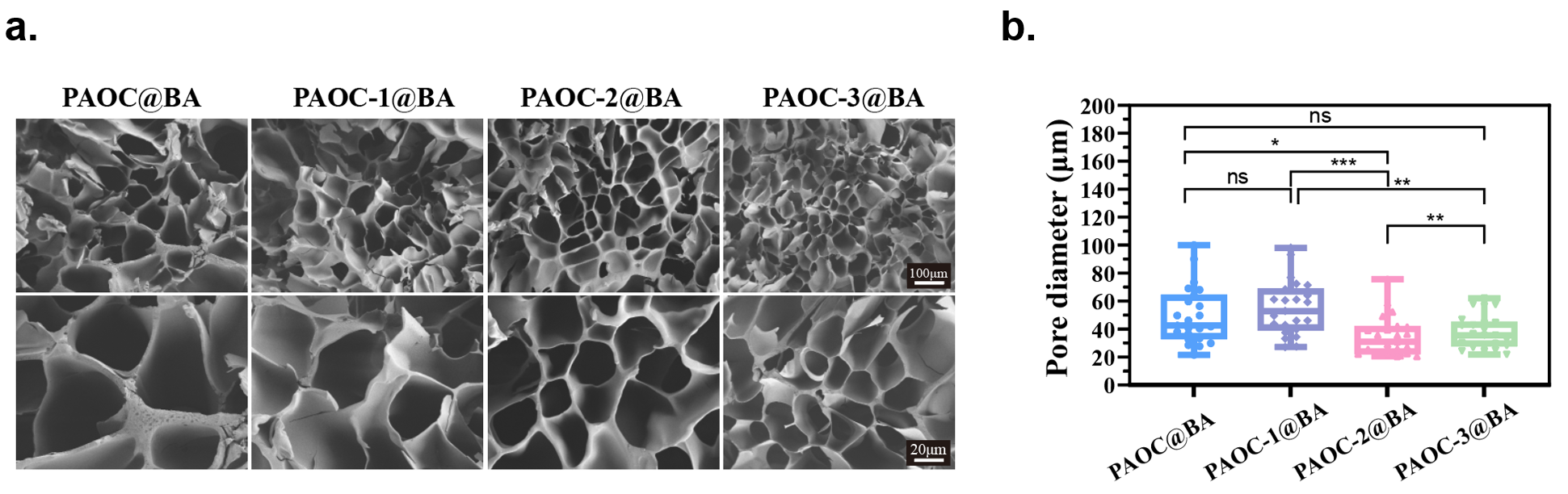


**Figure S10.** a-b) Scanning electron microscope images(a) of the solid-state of different hydrogels and the measured pore size (b) of the PAOC@BA hydrogel.


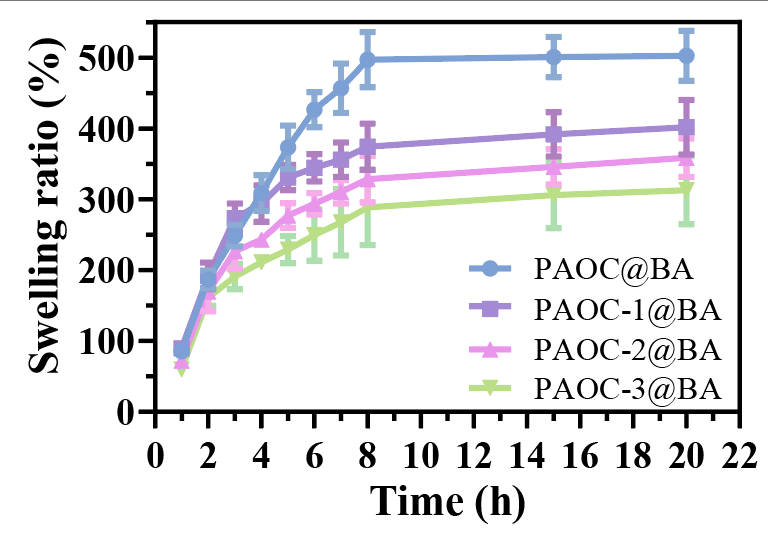


**Figure S11.** Real-time swelling rate of PAOC@BA hydrogel within 20 hours at 25 °C.


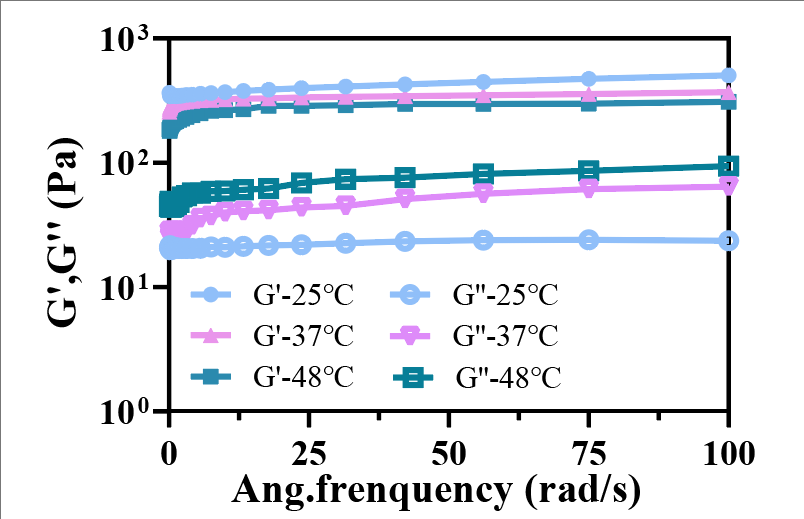


**Figure S12.** Rheological properties of PAOC@BA hydrogel at different temperatures (25, 37, and 48 °C) as a function of frequency (0.1~100 rad s-1).


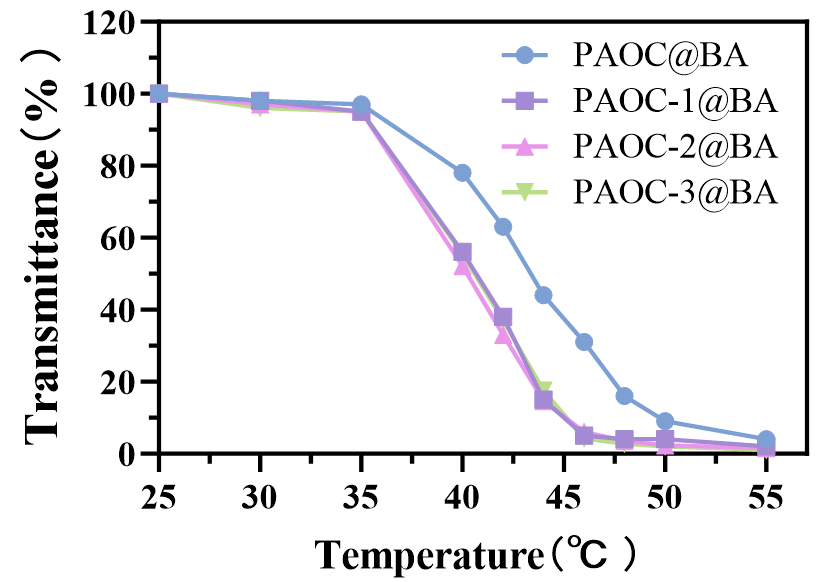


**Figure S13.** Transmittance of the different PAOC@BA hydrogels at 808 nm as a function of temperature.


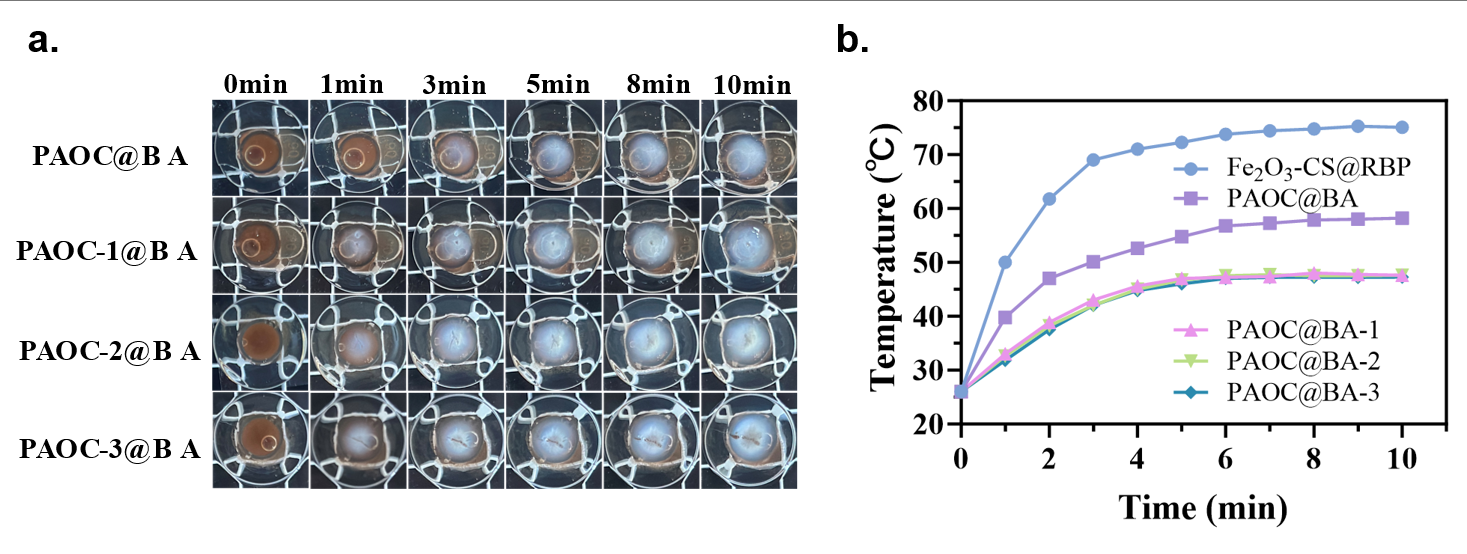


**Figure S14.** a-b) Photographs (a) and photothermal curves (b) showing the change of different groups of hydrogels after being irradiated by 808 nm NIR (1 W cm^-2^) with Fe_2_O_3_-CS@RBP (0.5mg/ml) incorporated.


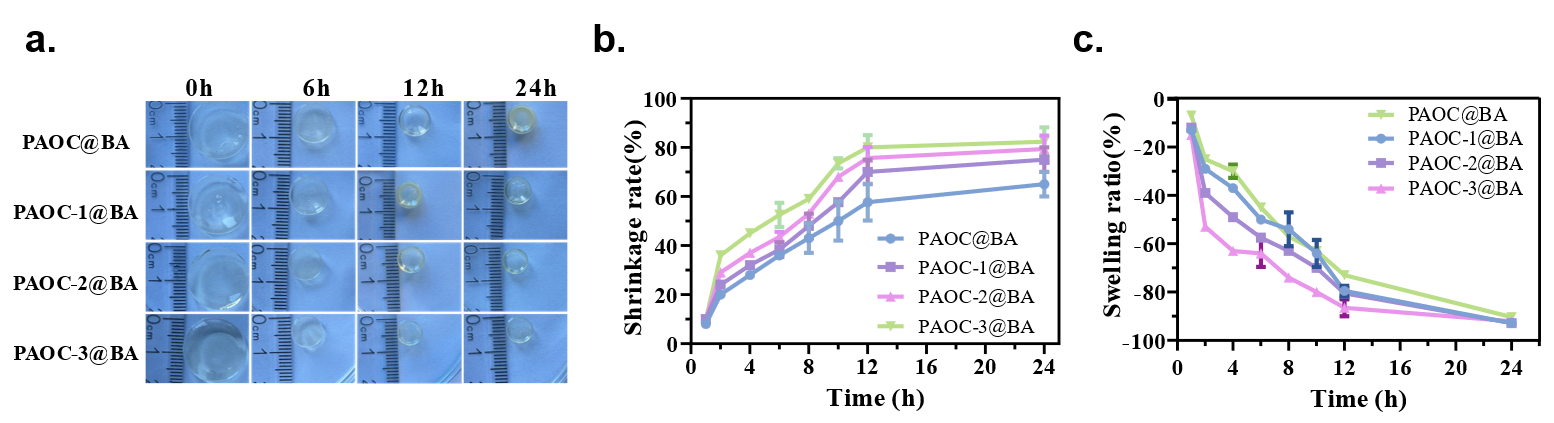


**Figure S15.** a-c) Photographs (a), the shrinkage rate (b) and the swelling rate (c) of the self-contraction of the different PAOC@BA hydrogels under thermal stimulation at 37 °C for 24 hours.


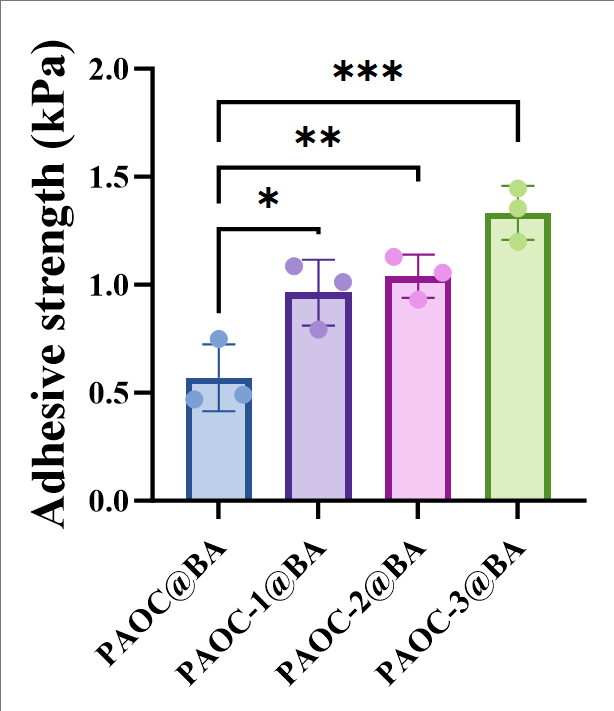


**Figure S16.** Adhesive strength of different groups of PAOC@BA hydrogels with consistent initial size at 25 °C.


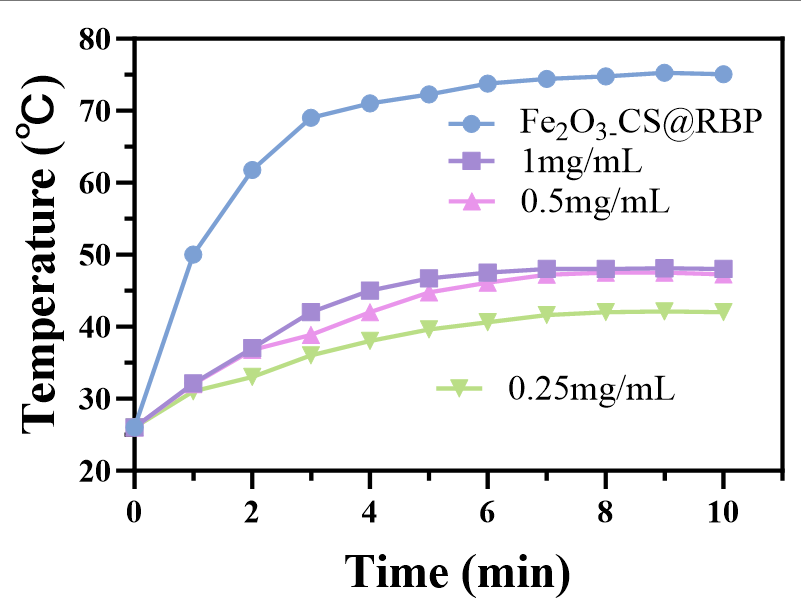


**Figure S17.** Photothermal curves of PAOC-3 hydrogel after being irradiated by 808 nm NIR (1 W cm^-2^) with different concentrations of Fe_2_O_3_-CS@RBP incorporated. The Fe_2_O_3_-CS@RBP represents the group of individual nanoparticles (0.5mg/mL).


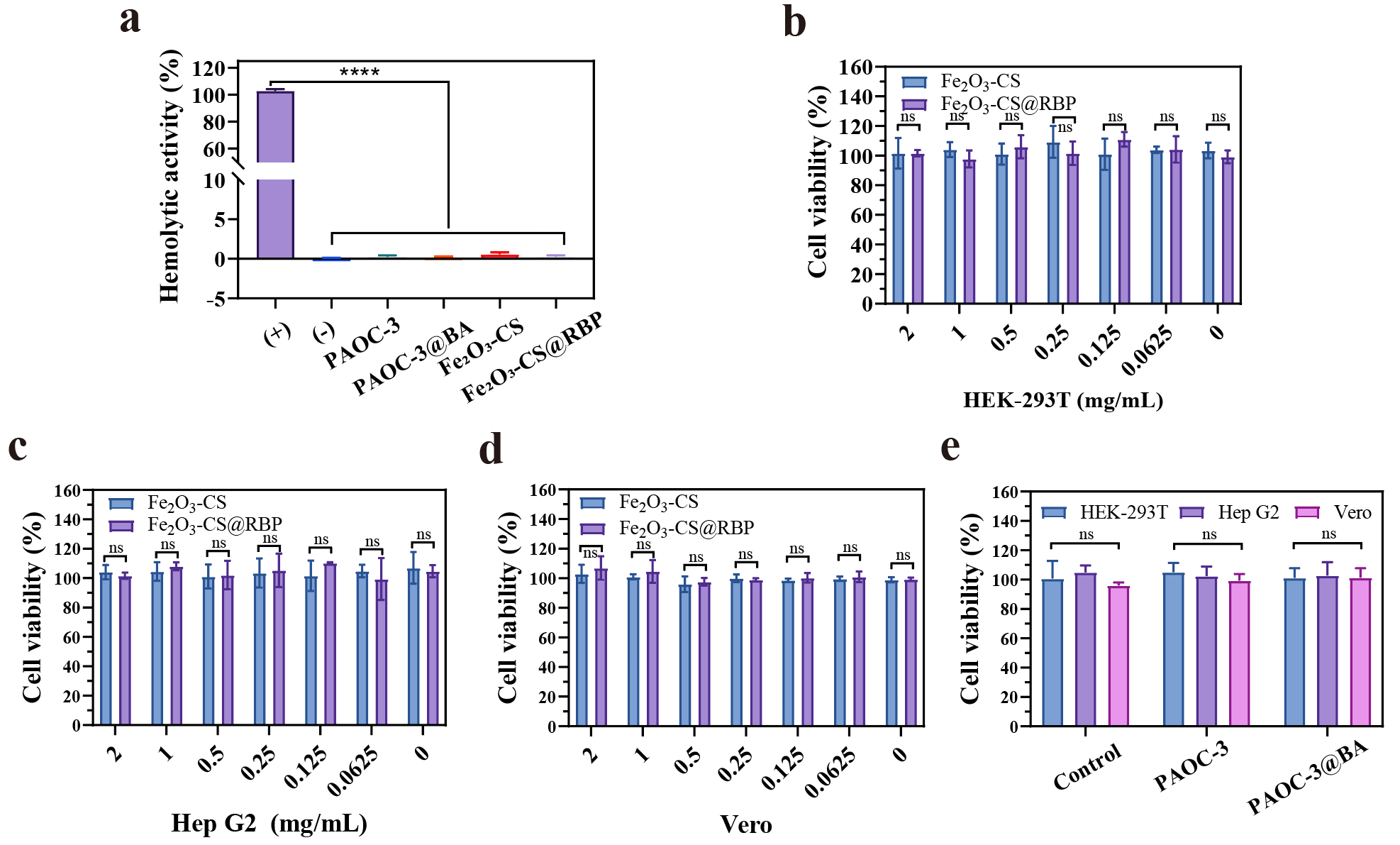


**Figure S18.** a) The hemolytic activity of Fe_2_O_3_-CS, Fe_2_O_3_-CS @RBP nanomaterials and PAOC, PAOC@BA hydrogel. Data are presented as mean ± standard deviation (n = 3 biological replicates). b-d) Cell viability of Vero cells, HEK-293T cells, and HepG2 cells after treatment with Fe_2_O_3_-CS, Fe_2_O_3_-CS @RBP NPs at concentrations ranging from 0.0625 to 2 mg/mL. e) Cell viability of Vero cells, HEK-293T cells, and HepG2 cells after treated with the hydrogel PAOC, PAOC@BA (14 mm in diameter, 2 mm in thickness).


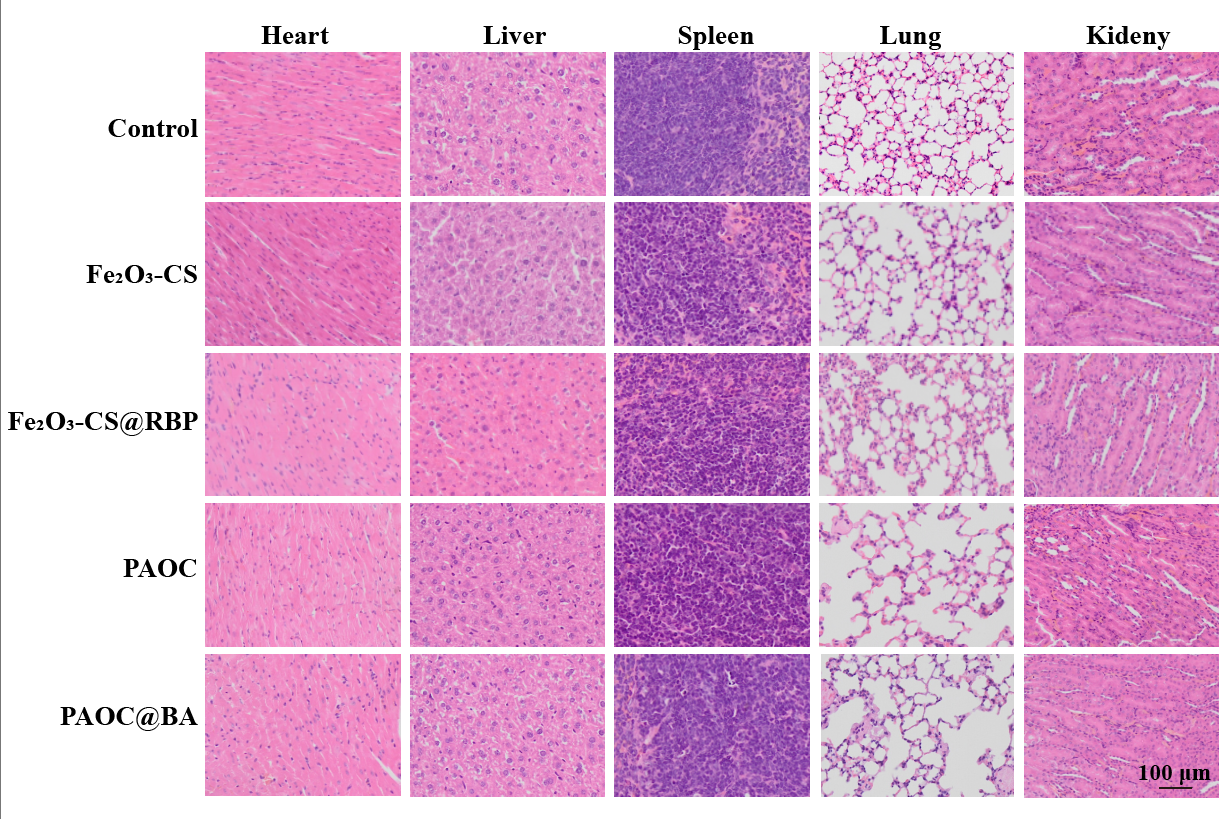


**Figure S19.** Histological Analysis: After in vivo treatment, paraffin sections of the main organ tissues of mice in different material groups were stained with hematoxylin and eosin. Independent experiments (n = 3 biological replicates) were performed with similar results.

**
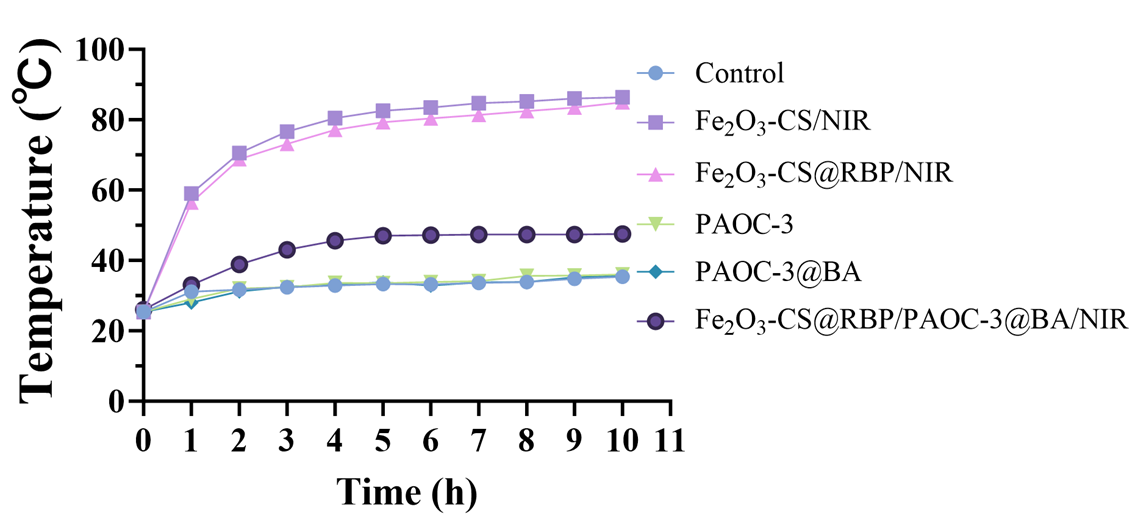
**

**Figure S20.** Wound area temperature in mice during different treatment.

**Table S1**: Gene sequence of RBP.

| Gene | Sequence |
| --- | --- |
| RBP | ATGGCGCTGAACTTTACCACCATTACCGAAAACAACGTGATTCGCGATCTGACCACCCAGGTGAACAACATTGGCGAAGAACTGACCAAAGAACGCAACATTTTTGATATTACCGATGATCTGGTGTATAACTTTAACAAAAGCCAGAAAATTAAACTGACCGATGATAAAGGCCTGACCAAAAGCTATGGCAACATTACCGCGCTGCGCGATATTAAAGAACCGGGCTATTATTATATTGGCGCGCGCACCCTGGCGACCCTGCTGGATCGCCCGGATATGGAAAGCCTGGATGTGGTGCTGCATGTGGTGCCGCTGGATACCAGCAGCAAAGTGGTGCAGCATCTGTATACCCTGAGCACCAACAACAACCAGATTAAAATGCTGTATCGCTTTGTGAGCGGCAACAGCAGCAGCGAATGGCAGTTTATTCAGGGCCTGCCGAGCAACAAAAACGCGGTGATTAGCGGCACCAACATTCTGGATATTGCGAGCCCGGGCGTGTATTTTGTGATGGGCATGACCGGCGGCATGCCGAGCGGCGTGAGCAGCGGCTTTCTGGATCTGAGCGTGGATGCGAACGATAACCGCCTGGCGCGCCTGACCGATGCGGAAACCGGCAAAGAATATACCAGCATTAAAAAACCGACCGGCACCTATACCGCGTGGAAAAAAGAATTTGAACCGAAAGATATGGAAAAATATCTGCTGAGCAGCATTCGCGATGATGGCAGCGCGAGCTTTCCGCTGCTGGTGTATACCAGCGATAGCAAAACCTTTCAGCAGGCGATTATTGATCATATTGATCGCACCGGCCAGACCACCTTTACCTTTTATGTGCAGGGCGGCGTGAGCGGCAGCCCGATGAGCAACAGCTGCCGCGGCCTGTTTATGAGCGATACCCCGAACACCAGCAGCCTGCATGGCGTGTATAACGCGATTGGCACCGATGGCCGCAACGTGACCGGCAGCGTGGTGGGCAGCAACTGGACCAGCCCGAAAACCAGCCCGAGCCATAAAGAACTGTGGACCGGCGCGCAGAGCTTTCTGAGCACCGGCACCACCAACAACCTGAGCGATGATATTAGCAACTATAGCTATGTGGAAGTGTATACCACCCATAAAACCACCGAAAAAACCAAAGGCAACGATAACACCGGCACCATTTGCCATAAATTTTATCTGGATGGCAGCGGCACCTATGTGTGCAGCGGCACCTTTGTGAGCGGCGATCGCACCGATACCAAACCGCCGATTACCGAATTTTATCGCGTGGGCGTGAGCTTTAAAGGCAGCACCTGGACCCTGGTGGATAGCGCGGTGCAGAACAGCAAAACCCAGTATGTGACCCGCATTATTGGCATTAACATGCCG |

**Table S2.** Iron release from nanoparticles over one week, quantified by using ICP-MS.

| Samples | *Fe* (mg/mg) | DMEM | |  | DMEM （Laser treatment） | |
| --- | --- | --- | --- | --- | --- | --- |
|  |  | *Fe* (μg/mL) | release rate |  | *Fe* (μg/mL) | release rate |
| Fe_2_O_3_-CS | 6.34×10^5^±  1.78×10^3^ | 2.34±0.03 | 0.19%±0.01% |  | 8.56±0.74 | 0.68%±0.06% |
| Fe_2_O_3_-CS@RBP | 4.54×10^5^±  1.77×10^3^ | 2.38±0.09 | 0.26%±0.01% |  | 8.84±0.65 | 0.97%±0.08% |

**Table S3**: Bacterial Strains and Cells Used in this study.

| Organism | Characteristics | Source |
| --- | --- | --- |
| *E. coli* | BL21(DE3), protein expression. | Thermo Fisher Scientific |
| *E. coli* | TOP10, plasmid construction, plasmid maintenance. | Thermo Fisher Scientific |
| *S. aureus* | ATCC43300, methicillin-resistant. | ATCC |
| *E. coli* | ATCC25922 | ATCC |
| *Vero* |  | ATCC |
| *RAW264.7* |  | ATCC |
